# A Clinically Applicable Unmixing Approach for Spectral MRD Detection in AML

**DOI:** 10.3390/cancers18142323

**Published:** 2026-07-18

**Authors:** Julian-Philipp Waclawski, Isabell Arnhardt, Hendrik Fokken, Nadine Kattre, Daphne den Hartog, Alexander N. Snel, Angele Kelder, Jessica Herbst, Martin G. Sauer, Michael Stadler, Michael Heuser, Costa Bachas, Adrian Schwarzer, Tobias Maetzig

**Affiliations:** 1 Department of Hematology, Hemostasis, Oncology, and Cell Therapy, Hannover Medical School, 30625 Hannover, Germany; 2 Department of Pediatric Hematology and Oncology, Hannover Medical School, 30625 Hannover, Germany; 3 Department of Hematology, Amsterdam UMC, Vrije Universiteit Amsterdam, 1081 HV Amsterdam, The Netherlands; 4 Cancer Center Amsterdam, Imaging and Biomarkers, 1081 HV Amsterdam, The Netherlands; 5 Department of Internal Medicine IV, University Hospital Halle (Saale), Martin-Luther-University Halle-Wittenberg, 06120 Halle, Germany; 6 Department of Internal Medicine C: Hematology, Oncology and Stem Cell Transplantation, University Medicine Greifswald, 17475 Greifswald, Germany; 7 Comprehensive Cancer Center Mecklenburg-Vorpommern, University Medicine Greifswald, 17489 Greifswald, Germany; 8 Institute of Experimental Hematology, Hannover Medical School, 30625 Hannover, Germany

**Keywords:** spectral flow cytometry, MRD diagnostics, AML, leukemic stem cells, hemodilution, viability assessment

## Abstract

**Background/Objectives**: Spectral flow cytometry enables the single-tube integration of all markers required for the assessment of measurable residual disease (MRD) in AML. The routine clinical use of spectral flow cytometry is challenged by the need for adequate single-stained unmixing controls. To pave the way for the clinical implementation of spectral flow cytometry, we opted to develop a multifunctional AML MRD panel, whose unmixing routine solely depends on ubiquitously available peripheral blood leukocytes (PBLs). **Methods**: We reconfigured our previously developed 19-color single-tube spectral MRD assay to incorporate viability, hemodilution, and leukemic stem cell (LSC) markers. In parallel, primitive markers were reassigned to non-tandem fluorochromes, resulting in a finalized 22-color panel. These fluorochromes were additionally leveraged as CD14 surrogate controls, whereas all other markers retained their dedicated fluorochromes for unmixing based on single-stained PBLs. **Results**: PBL-based controls enabled accurate spectral unmixing and reproducible assessment of CD16-based hemodilution and cell viability in spike-in experiments. Linear leukemia-associated immunophenotype (LAIP) detection, sensitivity, as well as intra- and inter-assay precision within predefined acceptance criteria were demonstrated in KG-1 dilution experiments using normal bone marrow (nBM). Furthermore, the 22-color assay resolved LAIPs and LSCs in patient samples with strong concordance to the 5-tube reference assay and revealed aberrant marker combinations that could not be assessed using the multi-tube design. **Conclusions**: This single-tube 22-color spectral flow cytometry assay may support a more clinically applicable and potentially standardizable approach to AML MRD diagnostics through a universal PBL-based unmixing strategy.

## Simple Summary

Acute myeloid leukemia (AML) is a hematological malignancy in which successful treatment depends on eliminating every remaining cancer cell. Sensitive laboratory tests are needed to detect even very small numbers of therapy-resistant cells, thereby providing important information for prognosis and treatment decisions. One such method, flow cytometry, uses fluorescent markers to identify abnormal leukemia cells. Newer “spectral” flow cytometers can measure even more markers at once, improving detection. However, a key challenge is generating suitable calibration controls to ensure accurate and reliable results. In this study, we developed an improved 22-color spectral flow cytometry test that uses readily available white blood cells from normal blood as simple practical controls. We show that this approach reliably detects residual AML cells and add functions to assess sample quality. Overall, this assay and its control strategy support the clinical use of spectral flow cytometry for detecting remaining leukemia cells.

## 1. Introduction

The treatment of acute myeloid leukemia (AML) typically consists of intensive induction chemotherapy, followed by consolidation therapy to prevent disease recurrence [1,2]. The presence of residual leukemic cells in the bone marrow (BM) following intensive induction therapy is associated with a poor prognosis and requires treatment, typically in the form of allogeneic stem cell transplantation [3,4]. Apart from RT-qPCR and next-generation sequencing, the flow cytometric assessment of MRD has become widely used due to its universal applicability and fast readout [5]. Multiparametric flow cytometry (MFC)-MRD identifies residual cells based on their aberrant cell surface protein expression. However, conventional flow cytometers are limited by the number of detectors and consequently the number of parameters that can be assessed simultaneously. Therefore, common antibody panels for MRD assessment on these flow cytometers require a multi-tube design, using a set of backbone markers (CD34, CD117, CD45, CD33, CD13 and HLA-DR) to identify the blast population in each tube, as well as unique markers to assess leukemic phenotypes [6,7]. As a result, other instrumental markers, e.g., for determination of cell viability or dilution of the BM sample with peripheral blood (PB) (e.g., CD16; hemodilution), are not commonly implemented in clinical diagnostic panels, although they could potentially contribute to quality assurance and enhanced functionality [5,6,7,8,9]. Furthermore, the multi-tube approach requires a large number of cells, which limits the availability of cell material for additional assessments such as the detection of LSCs. In particular, LSC detection requires relatively high cell numbers for providing a reliable read-out and has demonstrated prognostic relevance in the context of induction chemotherapy [10,11,12,13,14,15].

Recently, MFC-MRD panels for use with full-spectrum flow cytometers have been described that combine 19–29 antibodies in a single tube [16,17,18,19]. This has become possible due to the detection of the entire spectrum of fluorescence signals emitted by all antibody-fluorochrome conjugates [20,21]. As a main advantage of spectral flow cytometry, marker combinations that were previously distributed across multiple tubes can now be studied, and critical limits on the number of available cells are less of an issue. However, the large number of markers also poses challenges in terms of the accuracy of data acquisition, analysis and interpretation [22,23]. While assay design and validation traditionally focus on addressing these topics, spectral flow cytometry requires prior consideration of appropriate reference controls as early as the panel development stage. Such controls must express the corresponding antigens at sufficient frequency and density, enable the detection of rare fluorochrome–antigen combinations, share spectral properties with the target tissue, and ideally be prepared in a controlled and standardized manner. Although single-stained beads are commonly used as controls in conventional flow cytometry, they often fail to provide satisfactory spectral unmixing, possibly due to their altered autofluorescence and fluorochrome-dependent spectral signatures that differ from those of biological cells [24,25,26]. Moreover, whereas the emission spectra of non-tandem fluorochromes remain largely consistent across different antibody conjugates, tandem dyes exhibit subtle but relevant clone, conjugation, and lot-specific spectral variability. Consequently, reliable unmixing necessitates single-stained reference controls generated with the identical tandem fluorochrome-conjugated antibodies used throughout the panel. However, establishing such cellular reference controls is challenging, as accurate spectral reference generation requires adequate representation of all antigens included in the panel. To this end, we and others have successfully established reference controls based on nBM supplemented with leukemia cell-lines expressing rare hematopoietic markers, such as CD34, CD117, and CD133, thereby enriching the respective target populations to levels sufficient for the use of the same antibody–fluorochrome conjugates employed in the staining panel [17,19]. Although in general, cell-based controls appear to yield more accurate unmixing results [24,27,28], antibody capture beads, commercial reference controls, cellular preparations, and combinations thereof have also been explored as unmixing controls. However, each approach requires antibody-specific verification, which complicates standardization and hinders routine clinical implementation, making the identification of universally applicable reference controls a major practical challenge.

We have previously developed a 19-color spectral MFC-MRD assay that combines antibody clones from a clinically validated 5-tube panel into a single tube and demonstrates comparable sensitivity and accuracy for the detection of LAIPs [15,17]. We here report on the development of an improved 22-color panel in which a universally applicable PB-based unmixing strategy was embedded from the outset to overcome the limitations of conventional reference strategies. The resulting redesign expands the panel’s functionality and provides a clinically applicable unmixing approach that enables precise measurement of cell viability, hemodilution, and LSC burden alongside standard MRD analysis.

## 2. Materials and Methods

### 2.1. Sample Preparation and Staining

Pseudonymized BM samples from donors with informed consent were processed and stained as previously described using between 1 and 20 × 10^6^ cells per tube and resuspended in 200 µL buffer (100% PBS + 0.1% HSA) for acquisition [15,17]. At least 1 × 10^5^ cells were used as an unstained control and resuspended in 100 µL buffer for acquisition. Single-stained controls were based on pooled and lysed PB samples from anonymous healthy donors, of which 2.5 × 10^5^ cells were used for each single-stained control and were resuspended in 50 µL of buffer for acquisition. The 22-color antibody mixtures were prepared as specified in Table 1 and used for staining of samples in a total volume of 150 µL (incl. 10 µL of Brilliant Stain Buffer Plus (BD Biosciences; San Jose, CA, USA) and 48 µL of PBS; cells resuspended in 50 µL of buffer) for 15 min in the dark, before washing and resuspension for acquisition. For single-stained controls, the same antibody amounts (incl. 10 µL of Brilliant Stain Buffer Plus for each single stain; cells resuspended in 50 µL of buffer) and staining procedure were used. For primitive markers (CD34, CD117, and CD133) that are present at low frequencies or absent in PB, we used CD14 conjugates as surrogate markers with the corresponding non-tandem fluorochromes (Table 1). For single-stain controls of the Zombie NIR viability dye (BioLegend, San Diego, CA, USA) cells were heated to 95 °C for 5 min prior to staining to facilitate the separation of marker-positive and -negative cells. All 22-color measurements were performed on a 3-laser (V-B-R) Northern Lights CLC with CE-IVD certification (Cytek Biosciences; Fremont, CA, USA). Samples that were stained with the 5-tube assay were processed as previously described and recorded on a BD FACS Canto II (BD Biosciences; San Jose, CA, USA) [15]. An overview of all samples included in this study can be found in Table S1.

### 2.2. Limiting Dilution Experiments

Representative hemodiluted AML MRD samples were simulated by mixing nBM with 0.3% KG-1 cells (DSMZ no.: ACC 14) for spiking with PB dilutions of 10%, 20% and 40% of the corresponding BM donor, as adapted from Loken et al. [29].

For the assessment of conventional LAIP detection, nBM was spiked with the KG-1 cell-line in frequencies of 0.5%, 0.1%, 0.05%, 0.025%, 0.015%, 0.010%, 0.005%, and 0%. The samples were measured in two separate runs, with the quality control at the Northern Lights CLC repeated between them. 2 × 10^6^ cells were stained for all dilution experiments.

All dilution steps for hemodilution and LAIP-detection were stained in triplicates to assess intra-assay variability. Also, with each of these experiments, pure nBM samples (in triplicates) were measured to verify the absence of the KG-1 LAIPs. LAIP contents in the dilution steps were defined as following: expected LAIP content = LAIP content in the KG-1 cell line times the dilution step percentage, measured LAIP content = WBC- or PM-MRD frequency determined via gating using Infinicyt™ (Cytognos, S.L., Salamanca, Spain; 2.0.4.a.009-2020-02-01) software. The determination of linear regressions is referred to the data of expected LAIP content and not the percentage indicated by the dilution step, which is always at a higher percentage than the expected LAIP content, because the KG-1 cell line is split up in subpopulations, which do not overlap fully in their LAIP expression (Table S2). An overview of the analytical performance characteristics assessed in each experiment is provided in Table S3.

To test the performance of the assay on patient samples, AML samples from diagnosis were spiked into healthy bone marrow in dilutions of 1:10, 1:50, 1:250 and 1:1250 and subsequently stained and measured on the Cytek Northern Lights CLC (22-color panel) and the FACS Canto II (5-tube panel).

For hemodilution, LAIP detection as well as spike-in experiments for AML samples from diagnosis, distinct healthy bone marrows were used.

### 2.3. Data Acquisition, Spectral Unmixing, Post-Processing and Analysis

All samples were measured and analyzed on a Cytek Northern Lights CLC with a 3-laser (V16-B14-R8) configuration and SpectroFlo CLC 1.02. Instrument performance was checked daily through QC bead recordings. Samples were run at medium speed. Spectral unmixing was performed on a surrogate assay utilizing primitive marker-associated fluorochromes (CD34-BV421, CD117-PE, and CD133-APC) as CD14 conjugates for single-stained PB sample controls. The controls and corresponding samples were collected within two weeks of each other.

Accurate unmixing was defined as post-unmixing compensation adjustments remaining within ±3%. The manual compensation procedure was described in Fokken et al. [17]. In short, NxN plots for all 22 markers were compensated sequentially and repetitively for each sample until a normal marker profile was established. For validation experiments with dilutions of the material of the same patient, the compensation was only performed on the raw patient material (no spike-in) and then applied for all subsequent dilution steps. In line with current recommendations [23], exported fcs files were run through PeacoQC (as a FlowJo plugin) for removal of “bad” events characterized by, e.g., flow inconsistencies or anomalies in fluorochrome detection [30]. Subsequent analyses were done on exported “good” events in Infinicyt or FlowJo (BD). Likewise, UMAP and FlowSOM were run as FlowJo plug-ins [31,32]. The Infinicyt analyses of the limiting dilution experiments described in Section 2.2 were performed utilizing gating templates, that capture the gating of WBCs, blasts, CD34^+^ cells and the LAIPs of the KG-1 cell line, as shown in Figure S1. Gating templates were defined separately for each dilution experiment (hemodilution and LAIP-detection).

## 3. Results

### 3.1. Panel Development

The objective of the current study was to redesign our previously published 19-color panel to permit the use of universally accessible unmixing controls and to expand its analytical capabilities to include viability, hemodilution, and LSC content [17]. This required the shuffling of several antibody–fluorochrome combinations, while simultaneously permitting the addition of the most prevalent antibodies—CD123 and CD45RA—for the detection of LSCs [11], as well as CD16 for the assessment of hemodilution [29], and Zombie NIR for dead cell exclusion. CD16 was selected among published hemodilution markers due to its availability as a EuroFlow clone in multiple fluorochromes, enabling seamless integration into panel design and analysis workflows [29,33,34,35,36,37]. Furthermore, CD5 was omitted from the revised panel because of its limited specificity for AML-associated immunophenotypes [38]. These adaptations led to the multifunctional 22-color spectral MFC-MRD panel (Table 1). Importantly, the redesign of the panel was done by maintaining the clinically validated antibody clones that have been extensively used by the EuroFlow community [15,39]. Investigation of spectral properties showed limited overlap, resulting in a moderately low complexity index of 8.19 for the complete panel (Figure S2A). The highest spectral similarities were predicted for BV785 vs. BV750 = 0.81, APC-R700 vs. Spark NIR 685 = 0.79 and APC-Fire 810 vs. APC-Fire 750 = 0.76. Furthermore, the spectral plot revealed “open spaces” that could accommodate at least four additional fluorochromes as future drop-in markers (Figure S2B).

### 3.2. Establishment of Optimal Single-Stained Controls Based on PB and a CD14 Surrogate Approach

The major challenge in implementing complex marker panels is the establishment of appropriate reference controls. As PBL are positive for most MRD markers and exhibit autofluorescence characteristics comparable to those of the target populations (Figure S3A), they were selected as the primary reference matrix. However, the primitive markers CD34, CD117, and CD133 are largely restricted to bone marrow populations. Consequently, we investigated a surrogate-based approach for spectral unmixing control generation. In this strategy, antibodies conjugated to the fluorochromes used in the final panel were redirected to highly expressed alternative antigens, enabling the acquisition of sufficient positive events for high-quality reference controls. To establish suitable surrogate controls, we sought to identify a PB cell population that was both abundant and exhibited an autofluorescence profile comparable to that of bone marrow blasts. Monocytes fulfilled these criteria, resembling the blast autofluorescence spectrum (Figure S3A). On monocytes, CD14 was chosen as the surrogate target, owing to its uniformly high expression and the availability of EuroFlow-validated antibody clones (M5E2/MφP9) conjugated to the bright monovalent fluorochromes BV421, PE and APC.

Thus, following antibody titration and determination of optimal stain indices (Table S4), PB was single-stained with CD14-BV421 (instead of CD34), CD14-PE (instead of CD117), and CD14-APC (instead of CD133), as well as with the remaining antibodies of the 22-color panel (Table 1). However, despite the use of titrated antibodies, data analysis was hampered by a relatively strong spread between PE-Fire810 (HLA-DR) and PE (CD117) as well as BV785 (CD13) and between PE-CF594 (CD7) and BV605 (CD64) as evident in NxN plots (Figure S4A). Careful examination of the data suggested that the cause of these problems were overly bright signals, which were resolved by further dilution of the HLA-DR (1:25 -> 1:400) and CD7 (1:100 -> 1:800) antibodies (Figure S4B,C). Collectively, these changes improved signal resolution in fully stained nBM (Figure S4A,B).

Next, we opted to compare the performance of the PB-based surrogate unmixing approach with a conventional unmixing approach using a mixture of nBM and cell lines [17]. For both approaches, the unmixing matrices remained within the predefined accuracy threshold (±3%) and showed only minimal differences (Figure S3B). This indicates that spectral residuals and unmixing errors were detected in largely the same fluorochromes and were quantitatively acceptable, according to our predefined acceptance criterion. In particular, side-by-side comparison of the unmixed and compensated NxN plots for major WBC populations using representative population-specific markers, including CD4 for lymphocytes, CD14 for monocytes, and CD16 for granulocytes, as well as dim and primitive markers, including CD64, CD34, CD117 and CD133, showed strong similarity between the two approaches (Figure S3C–F).

**Figure 1:**
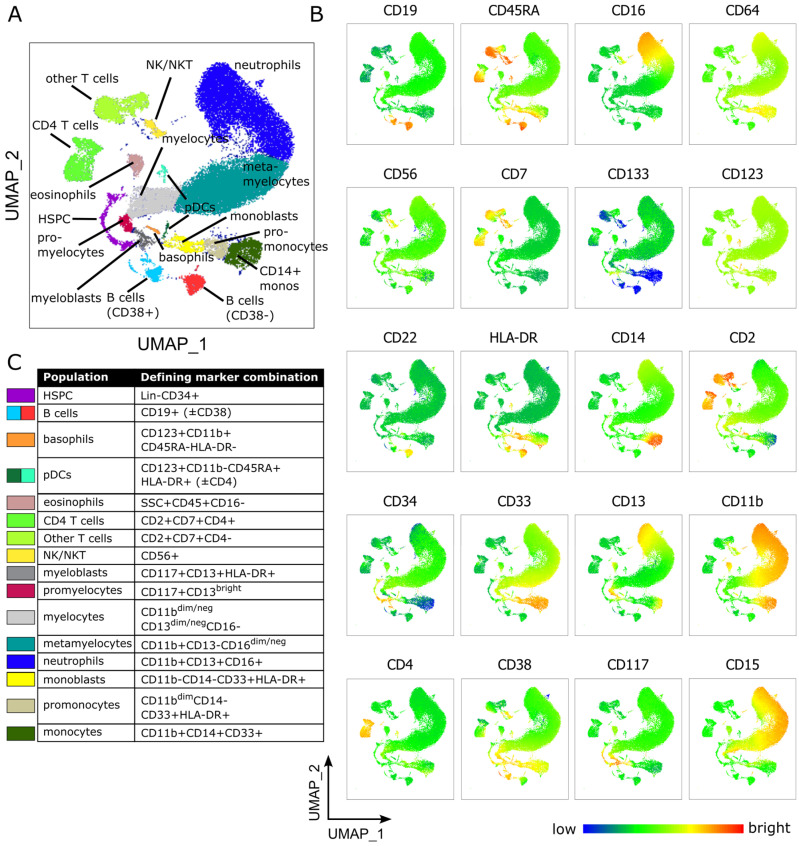
**The 22-color MFC-MRD panel resolves normal hematopoietic bone marrow populations.** (**A**) UMAP representation of a stained normal bone marrow sample. Clusters were identified by FlowSOM (20 metaclusters; 20 × 20 grid size) analysis prior to manual annotation based on marker expression profiles. The promyelocyte cluster was added retrospectively based on its bright CD13 signal. (**B**) Heat map representation of marker expression profiles. (**C**) Marker combinations for cell type annotations. Color codes are identical to A. Data analysis was performed on 5 × 10^4^ downsampled normal bone marrow cells, gated as viable CD45^+^ singlets. CD45 and Zombie NIR were thus not considered for clustering, FlowSOM and heat map representations.

Finally, PB-derived reference controls enabled accurate unmixing of a fully stained nBM sample, thereby qualifying the surrogate-based unmixing approach (Figure S5). Consequently, reproducible detection of all important normal bone marrow cell populations was achieved in the high-dimensional data space (Figure 1) and supported the tracking of representative differentiation trajectories in 2-dimensional plots (Figure S6).

### 3.3. The Addition of CD16 and Zombie NIR Improve the Quality of the Assay

**Figure 2:**
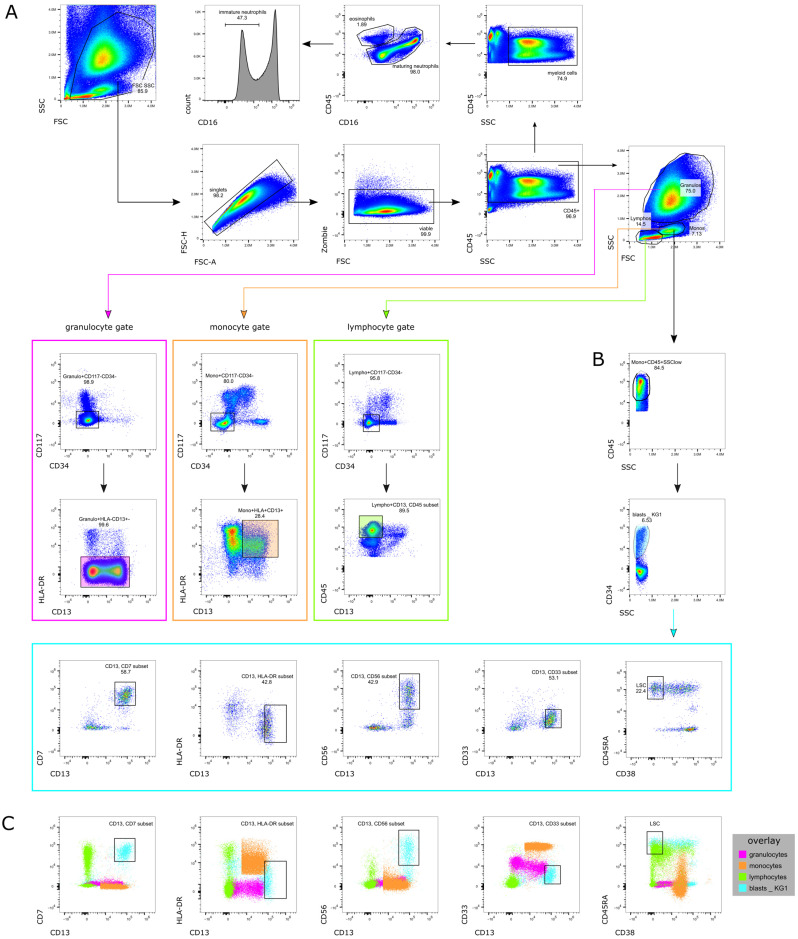
**Representative gating strategy for the identification of KG-1 derived blasts.** Bone marrow was spiked with KG-1 cells and subsequently stained with the 22-color spectral MFC-MRD panel for the identification of blasts. (**A**) Gating strategy for the identification of mature CD16^+^ granulocytes for assessment of hemodilution, pan granulocytes (SSC^high^FSC^high^CD117^−^CD34^−^HLA-DR^−^; pink), monocytes (SSC^dim^FSC^high^CD117^−^CD34^−^HLA-DR^+^CD13^+^; orange), and lymphocytes (SSC^low^FSC^low^CD117^−^CD34^−^CD45^bright^CD13^−^; green) after gating for single, viable cells. (**B**) Identification of blast-like KG-1 cells within the SSC^dim^FSC^high^CD45^+^CD34^+^ gate. LAIPs are specified by subsequently gating for CD7^+^CD13^+^, HLA-DR^−^CD13^+^, CD56^+^CD13^+^, CD33^−^CD13^+^, and CD45RA^+^CD38^−^ (LSC). (**C**) Overlap of the granulocytes (pink), monocytes (orange), lymphocytes (green) and blasts (teal). LAIPs are indicated.

To evaluate the applicability of the PB-based unmixing strategy for standardized MRD diagnostics and to validate the additional functionalities of the 22-color MFC-MRD assay, we used nBM spiked with the leukemia cell line KG-1. The KG-1 cell population formed a distinct cluster within the nBM in the UMAP representation (Figure S7A–E) and displayed four conventional LAIPs (CD34^+^ with CD13^+^CD7^+^, CD13^+^CD56^+^, CD13^+^CD33^−^, and CD13^+^HLA-DR^−^) as well as an additional LSC LAIP (CD34^+^CD38^−^ CD45RA^+^) (Figure 2 and Figure S7F). These results suggest that the PB-based unmixing approach could also work in the MRD scenario. Using this experimental setup, we assessed intra- and inter-assay reproducibility, as well as assay specificity, as described previously [17,40,41].

**Figure 3:**
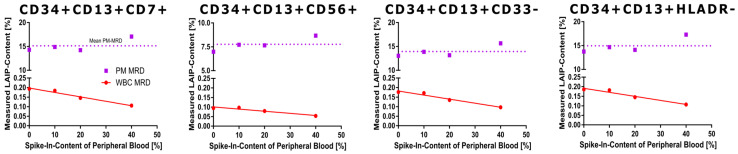
**Influence of hemodilution on LAIP frequencies.** Hemodilution was mimicked by increasing contributions of PB to a bone marrow KG-1 (0.3%) cell mixture, and subsequent assessment of the LAIP frequencies. For n = 12 samples (4 triplicates), linear regression of the resulting LAIP-frequencies is shown in red. PM-MRD results of the hemodiluted samples are shown in violet. = 12 samples (4 triplicates), linear regression of the resulting LAIP-frequencies is shown in red. PM-MRD results of the hemodiluted samples are shown in violet.

Next, a mixture of nBM and 0.3% KG-1 cells (BM^KG^), simulating an MRD-positive sample, was mixed with increasing amounts of PB of the same donor to resemble a hemodilution scenario, which in a clinical context is caused by repeated pulls during bone marrow aspiration. This was reflected by increasing amounts of mature CD16^+^ neutrophils, as well as decreasing amounts of immature CD16^dim^ neutrophils with increasing dilution steps (Figure S8A) [29]. Assessing WBC-MRD in the hemodiluted samples demonstrated that increasing amounts of PB led to a substantial underestimation of LAIP content (Figure 3). Notably, this could be overcome by using primitive marker (PM-) MRD, which, in accordance with Tettero et al. [40,42], yielded stable LAIP frequencies regardless of PB contamination (Figure 3) [8,9]. Mean values and standard deviation for these four conventional LAIPs were CD34^+^CD13^+^CD7^+^: 15.1 ± 1.6%, CD34^+^CD13^+^CD56^+^: 7.8 ± 0.8%, CD34^+^CD13^+^CD33^−^: 13.9 ± 1.5%, CD34^+^CD13^+^HLA-DR^−^: 14.9 ± 1.7%.

In summary, the addition of CD16 enabled the identification of hemodiluted samples for reporting and subsequent assessment by PM-MRD.

Furthermore, we investigated the applicability of Zombie NIR as a viability marker in our panel. To do so, we compared the number of WBCs when gating with and without this marker. We found only a difference of about 1% between the two gating strategies (Figure S8B). This indicates that standard scatter characteristics suffice to eliminate the majority of dead cells from the analysis in fresh samples.

### 3.4. The 22-Color MFC-MRD Assay Meets Important Quality Criteria for Clinical Use

**Figure 4:**
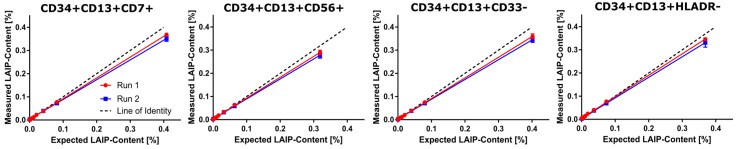
**Detection capability, precision and linearity of LAIP assessment.** Limiting dilution spike-in of KG-1 cells into nBM was used to determine the linearity of LAIP detection via linear regression analyses in two separate runs. These datasets were also used to determine the LoB, LoD, LoQ and CV. For details on CV, LoB, LoD and LoQ values see Tables S5 and S6.

When assessing MRD, assay dependent factors (e.g., machine, type and number of fluorochromes, antibody clones) may influence the sensitivity and specificity of MRD assessment and ultimately a reliable MRD result. Strategies for adequate sample unmixing are therefore of paramount importance, as they influence all downstream analyses. Consequently, using the PB-based unmixing strategy, we aimed to determine the limits of blank (LoB), detection (LoD), and quantitation (LoQ), as well as intra- and inter-assay variability, quantified through the coefficient of variation (CV), for our 22-color MRD assay, as important benchmarks for the specificity, sensitivity, and reproducibility of the assay [43]. To this aim, we spiked KG-1 cells in nBM in a range of proportions (0.005–0.5%) (Tables S2 and S3). Notably, in this set of data (n = 36), PeacoQC eliminated an average of 10.1 ± 7.5% of cells as “bad” events (Figure S9). Regardless, the dilution series with KG-1 cells demonstrated the unequivocal detection of the four conventional LAIPs down to a minimal limit of quantitation (LoQ) of 0.008% (CD34^+^CD13^+^CD7^+^) within the CD45^+^ BM fraction (Figure 4 and Table S5). This complies with the required sensitivity of 0.01% for MRD detection when using 0.1% as the diagnostic cut-off for positivity, as suggested by the European LeukemiaNet MRD Working Party [35]. The expected and the measured cell concentrations were highly correlated in linear regression analyses (R^2^ > 0.98) for all four conventional LAIPs, demonstrating the linearity of the assay (Figure 4).

Moreover, for the CV in intra- and inter-assay experiments we imposed an ideal limit of 10% and an acceptable limit of 25% [40,41,44,45]. The ideal limit could not always be met, but all CV values of LAIP-dilution and hemodilution experiments are <20% and therefore acceptable (Table S6). Because this analysis was done through a standardized gating template (Figure S1), the results underscore the reproducibility of the 22-color assay.

In summary, our performance values met the critical benchmarks required for clinical use of the 22-color MFC-MRD assay as listed in Tables S5 and S6, which further underlines the applicability of the PB-based unmixing approach.

### 3.5. WBC-MRD Is Concordant Between Conventional and Spectral Flow Cytometry in Spike-In Assays

**Figure 5:**
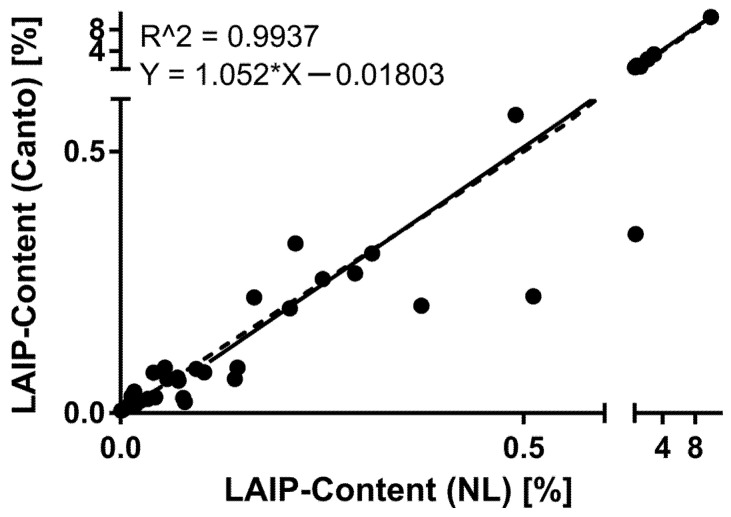
**High correlation of LAIP contents in limiting dilution experiments of AML diagnosis samples in nBM between the 5-tube assay and single-tube assay.** First-diagnosis AML samples were spiked into healthy bone marrow at dilutions of 1:10, 1:50, 1:250, 1:1250 to assess LAIP detection in patient-derived samples and compare performance with the current gold standard MFC-MRD approach (5-tube panel analyzed on a FACS Canto II). WBC-MRD results of the respective LAIPs have been concatenated and used for linear regression analysis. The dotted line represents the expected results.

Having demonstrated the advanced functionality of the 22-color spectral assay, we next compared its clinical applicability and specificity to the current 5-tube gold standard assay run on a conventional FACS Canto II cytometer [15,40]. Of note, this comparison involves the application of two distinct, instrument-specific unmixing/compensation strategies, based on PB (Northern Lights CLC) and beads (FACS Canto II) respectively. To this end, we spiked healthy bone marrow (n = 4) with AML patient samples from the timepoint of diagnosis (n = 3) at dilutions of 1:10, 1:50, 1:250 and 1:1250 and measured aliquots of the identical samples on the same days with the conventional and spectral flow cytometers. WBC-MRD analyses identified 1–6 concordant LAIPs per patient and assay format (CD34^+^CD13^+^CD7^+^ (n = 1), CD34^+^CD13^+^HLA-DR^−^ (n = 1), CD117^+^CD13^+^CD7^+^ (n = 2), CD117^+^CD13^−^CD33^+^ (n = 1), CD117^+^CD13^+^CD33^−^ (n = 1), CD117^+^CD13^+^HLA-DR^−^ (n = 2), CD133^+^CD34^−^ (n = 1) and CD133^+^CD13^+^HLA-DR^−^ (n = 1)). For all LAIPs, both assays returned comparable frequencies at each dilution step. Linear regression analysis of all data points showed an R^2^ >0.99 and a slope of 1.052 (Figure 5, Table S7).

### 3.6. MRD Results Are Concordant Between Conventional and Spectral Flow Cytometry in Fresh and Frozen AML Samples

**Figure 6:**
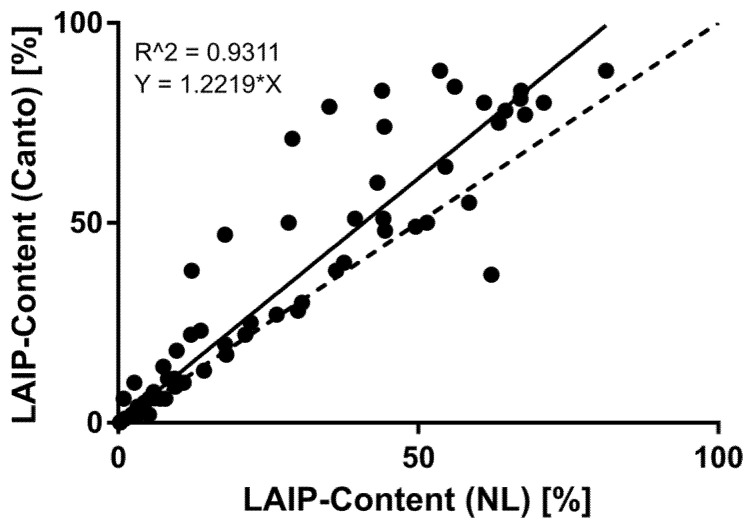
**High correlation of LAIP contents between the 5-tube assay and the 22-color single-tube assay in fresh and frozen AML MRD samples.** Diagnostic AML MRD patient samples were measured with the 5-tube panel on a BD FACS Canto II, while frozen aliquots of these samples were assessed with the 22-color single-tube assay on the Cytek Northern Lights CLC. Analysis of the data was performed via manual gating in Infinicyt. Each datapoint is defined through the results of LAIP/LSC-Content analysis of the corresponding LAIPs in corresponding measurements. LAIP/LSC-Content was defined by PM-MRD analysis.

To further assess the robustness of our assay and unmixing approach, we compared LAIP identities and their frequencies (expressed as a proportion of the respective primitive marker positive blast compartment; PM-MRD) between the conventional 5-tube panel (FACS Canto II) applied to fresh samples and frozen aliquots from the same patients and time points (n = 18) stained with our novel single-tube 22-color panel (Northern Lights CLC). Notably, WBC LAIP/LSC percentages are not meaningful in this context, because, e.g., granulocytes usually do not survive the freezing and thawing process, influencing the total WBC count significantly. In total, the PM-MRD analysis yielded 84 datapoints, which consisted of 26 distinct LAIPs and 4 different LSC phenotypes (Figure 6, Table 2). Notably, the 22-color panel captured 6 unique LAIPs, which were not detected in the 5-tube panel, due to the distribution of the relevant markers across different tubes. This underlines the obvious advantage of single-tube high-complexity antibody panels for MFC-MRD. However, we also observed discrepancies between the two assay formats for two samples. In one sample, two LAIPs (CD34^+^CD13^+^CD22^+^ and CD34^+^CD13^+^CD56^+^) could not be detected using the 22-color panel, even though these LAIPs were captured in other samples. Moreover, in another sample, we observed a systematic drop of all LAIP sizes from the fresh to the frozen sample. Since cryopreservation-associated differences in LAIP frequencies have been reported previously [46], and the source of the substantial discrepancies observed in our dataset could not be resolved, the corresponding samples were excluded from the regression analysis. Regardless, using the remaining 70 data points, we performed a linear regression comparison (centered at the origin), which yielded an R^2^ = 0.93 (slope = 1.22).

### 3.7. MRD Results Are Concordant Between Conventional and Spectral Flow Cytometry in Clinical AML Samples

To test our panel under clinical conditions, we performed a side-by-side comparison of five fresh AML MRD samples stained with the 5-tube gold-standard assay and the 22-color panel. Spectral unmixing with the PB surrogate approach showed consistent and accurate unmixing results (comparable to Figure S5).

**Figure 7:**
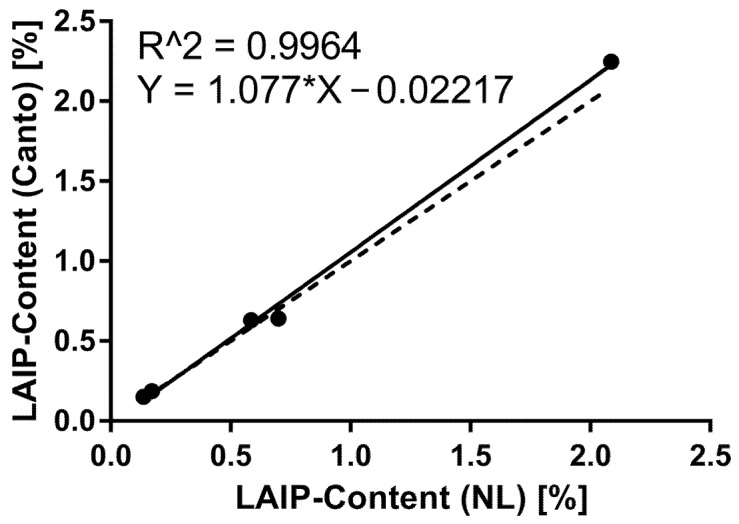
**High correlation of LAIP contents between the 5-tube assay and the 22-color single-tube assay in fresh AML MRD samples.** Fresh diagnostic AML MRD patient samples were measured with the 5-tube panel on a BD FACS Canto II and with the single-tube 22-color assay on the Cytek Northern Lights CLC. Analysis of the data was performed via manual gating in Infinicyt.

Importantly, MRD classifications were fully concordant across all five samples, with two classified as MRD-negative and three as MRD-positive (Figure 7 and Table S8). The MRD-positive samples comprised four distinct LAIPs characterized by dim and heterogeneous antigen expression. Even under these challenging conditions, LAIP frequencies showed strong concordance with those obtained using the 5-tube gold-standard panel. These findings extend and complement the results of the KG-1 dilution experiments, based on homogeneously bright LAIPs (Figure 4) and demonstrate the robustness of the assay across diverse antigen expression patterns.

Furthermore, hemodilution was evaluated in these five AML samples using the fraction of mature CD16^+^ neutrophil granulocytes, with a predefined cutoff of 30% [29]. Only one of the five samples exceeded this threshold (Figure S10), indicating significant hemodilution. As this hemodiluted sample was MRD-negative in both assays, first clinical MRD interpretation should be used with caution, second PM-MRD should be performed in addition to WBC-MRD, and if possible, third the bone marrow aspiration should be repeated to obtain a precise measure of MRD.

Taken together, these results show that implementing a PB-based unmixing strategy in the newly designed 22-color panel yields specificity and sensitivity comparable to the 5-tube panel, while expanding functionality and the detection of LAIP combinations beyond those identifiable by the conventional assay.

## 4. Discussion

The present study describes the development and technical validation of a multifunctional 22-color single-tube spectral flow cytometry assay for AML MRD assessment. In contrast to conventional multi-tube approaches [15], the assay combines established MRD markers with viability assessment, CD16-based evaluation of hemodilution, and LSC characterization within a single workflow (Table 1). At the same time, the panel architecture was designed with sufficient flexibility to accommodate future modifications. Additional marker–fluorochrome combinations can be incorporated without fundamentally changing the assay structure (Figure S2B), enabling adaptation to emerging MRD and LSC biomarkers such as TIM-3 and CLEC12A [11,13,47,48,49], or local laboratory requirements. Likewise, several antibody-fluorochrome assignments may be exchanged to match institutional preferences while preserving the overall assay concept [35].

A major technical challenge during panel development was the generation of robust spectral reference controls. Unlike conventional bead-based compensation, spectral unmixing relies heavily on high-quality single-stained controls and may be affected by differences in autofluorescence, signal intensity and fluorochrome signatures between biological cell sources and compensation beads [24,25,26].

In our previous work, satisfactory unmixing performance was achieved using controls based on nBM samples spiked with leukemic cell lines [17]. Although effective, such an approach is impractical for routine diagnostics and difficult to standardize across laboratories. To address this limitation, we explored a surrogate-based strategy using PB as a universally available cell source. Because primitive markers such as CD34, CD117, and CD133 are largely absent from PB, a CD14 surrogate approach was implemented that enabled the generation of robust single-stained reference controls for their respective non-tandem fluorochromes. This strategy substantially simplifies workflow implementation and eliminates the need for dedicated bone marrow reference material or leukemic cell lines [17,19]. Furthermore, laboratories with routine access to bone marrow samples may readily adapt the surrogate concept to bone marrow cells if concerns regarding tissue-specific autofluorescence arise [50].

Beyond control generation, reliable spectral MRD analysis requires dedicated measures to ensure data quality. Spectral flow cytometry introduces several potential sources of variability that are less prominent in conventional flow cytometry, including unmixing artifacts, autofluorescence-related effects, and spreading errors [50,51]. We therefore implemented a workflow that combines optimized unmixing controls with automated event-quality assessment and post-acquisition cleanup procedures, including PeacoQC-based removal of technically aberrant events [23,30,52]. In addition, minor sample-specific post-unmixing compensation adjustments were applied when required. While effective, such manual interventions remain time-consuming and require substantial operator expertise. Continued improvements in unmixing algorithms and automated compensation strategies therefore represent an important prerequisite for broader routine adoption of spectral MRD diagnostics [53]. Nevertheless, the integrated workflow presented here demonstrates that technically robust spectral MRD analysis can already be achieved using currently available tools and standardized quality control procedures (Figure 5, Figure 6 and Figure 7).

An important strength of the assay is its multifunctionality. Besides conventional LAIP-based MRD assessment, the panel incorporates several clinically relevant parameters that are often evaluated separately.

First, viability assessment was included through the use of Zombie NIR. Its contribution was modest in the predominantly fresh samples analyzed in this study, but the marker is expected to gain importance when analyzing stored, transported, or otherwise compromised specimens, where dead-cell accumulation may substantially affect data quality [40].

Second, CD16 was incorporated as a practical indicator of bone marrow hemodilution [29]. Assessment of hemodilution is increasingly recognized as an important component of AML MRD interpretation, as PB-contamination can lead to underestimation of residual disease levels [42]. Integration of CD16 therefore enables simultaneous evaluation of sample quality and MRD status within a single analytical workflow. However, it needs to be stressed that various markers have been explored to identify hemodilution, but none of them could so far be used to reliably correct MRD levels [42]. Therefore, quantitative adjustment for hemodilution regarding MRD assessment warrants further evaluation. This limitation can be overcome by reverting to PM-MRD instead (Figure 3) [42].

Finally, the panel includes markers required for LSC detection. LSC assessment remains exploratory both within the present study and in the broader AML community. In the current study, the limits of blank, detection, and quantitation were not formally assessed. Moreover, there is currently no consensus regarding the optimal threshold for defining clinically relevant residual LSC burden [10,14,54]. Nevertheless, we successfully identified LSC-associated phenotypes in post-treatment AML samples (Table 2), supporting the feasibility of concurrent MRD and LSC analysis using a single spectral assay.

Notably, clinical validation was performed in a limited number of AML MRD samples (n = 23), restricting conclusions regarding diagnostic performance in routine post-treatment bone marrow specimens. Nevertheless, the favorable results obtained in spike-in experiments, thawed and fresh AML MRD samples overall support the clinical potential of the assay. Future studies involving larger patient cohorts will therefore be required to determine the clinical significance and quantitative performance.

Looking beyond the present study, continued development of spectral MRD diagnostics will depend on both technological and organizational advances. An increasing number of computational tools are becoming available that incorporate spectral similarity, signal intensity, spreading characteristics, and marker-expression patterns during panel design [51,55]. These developments are expected to reduce the complexity of future panel optimization efforts [56]. Nevertheless, implementation of spectral MRD assays remains resource-intensive and requires expertise in unmixing, autofluorescence management, spectral quality control, and increasingly high-dimensional data analysis [22,23,56,57]. Consequently, the full benefits of spectral MRD assessment may not become broadly accessible until harmonized workflows and standardized analytical frameworks have been established.

Collaborative initiatives such as the ELN MRD Working Party will therefore play a crucial role in defining standardized marker backbones, analytical pipelines, and validation strategies for spectral AML MRD assessment, and should be supported by interlaboratory comparison studies and external quality-assessment programs [5,35,40]. For spectral flow cytometry specifically, harmonization of reagent batches, buffer formulations, instrument settings, and quality-control materials across participating centers will be particularly important. In addition, multicenter studies should systematically evaluate the impact of autofluorescence extraction on weak antigen detection, assess the utility of viability dyes under different sample-handling conditions, and determine the value of CD16-based hemodilution assessment in conjunction with PM-MRD reporting. The resulting insights will enable further refinement of marker combinations and analytical workflows and ultimately support robust, reproducible, and clinically applicable MRD diagnostics.

Until such harmonized approaches become available, additional spectral MRD panels will undoubtedly emerge and continue to evolve. Individual laboratories should therefore critically evaluate and adapt these approaches according to their local requirements and clinical settings. We anticipate that the design principles, quality-control strategies, and practical solutions presented here will contribute to these ongoing developments and support the long-term establishment of a standardized spectral AML MRD platform.

## 5. Conclusions

In this study, we developed and technically validated a 22-color single-tube spectral flow cytometry assay for AML MRD, while incorporating a practical peripheral blood-based unmixing strategy from the outset of panel design. The assay demonstrated robust analytical performance and strong concordance with a conventional 5-tube MRD assay while enabling detection of additional marker combinations. Collectively, these findings support the feasibility of a single-tube 22-color spectral flow approach and highlight its potential to facilitate future standardization of AML MRD diagnostics, pending broader clinical and interlaboratory validation.

## Figures and Tables

**Table 1:** Spectral MFC-MRD panel comparison.

Marker	Clone	19C Fluorochrome	22C Fluorochrome	µL per Test	Manufacture	Catalog Number
CD2	MT910	FITC	FITC	1.5	Dako	F076701-2
CD4	SK3	PE-Cy5	Spark Blue 550	1.5	BioLegend	344656
CD5	L17F12	BV480	n.a.	n.a.	n.a.	n.a.
CD7	M-T701	BV786	PE-CF594	0.2	BD	562541
CD11b	D12	BV750	BV750	1.0	BD	747210
CD13	WM15	BV421	BV785	4.0	BioLegend	301726
CD14	MφP9	BV650	BV650	2.0	BD	740633
CD15	MMA	PerCP-eF710	Pacific Blue	1.5	BioLegend	394704
CD16	3G8	n.a.	BV570	1.0	BioLegend	302036
CD19	SJ25C1	APC	PE-Cy5	1.5	BioLegend	363042
CD22	S-HCL1	PE	APC-Fire 750	6.0	BioLegend	363522
CD33	P67.6	APC-R700	APC-R700	4.0	BD	659122
CD34	8G12	BB700	BV421	0.2	BD	744904
CD38	HB7	APC-Fire 810	APC-Fire 810	2.0	BioLegend	356644
CD45	J.33	Krome Orange	Krome Orange	4.0	Beckman Coulter	B36294
CD45RA	HI100	n.a.	Spark-NIR 685	1.5	BioLegend	304168
CD56	MY31	BV711	PE-Cy7	1.5	Tonbo	60-0564-T100
CD64	10.1	BV605	BV605	2.0	BD	740406
CD117	104D2D1	PE-Cy7	PE	2.0	Beckman Coulter	IM2732
CD123	7G3	n.a.	BV480	1.0	BD	746673
CD133	AC133	PE-Vio615	APC	3.0	Miltenyi	130-113-106
HLA-DR	L243	APC-H7	PE-Fire810	0.4	BioLegend	307683
Viability	—	n.a.	Zombie NIR	0.15	BioLegend	423105
**Primitive marker unmixing controls**						
**Marker**	**Clone**	**19C** **Fluorochrome**	**22C** **Fluorochrome**	**µL** **per Test**	**Manufacture**	**Catalog** **Number**
CD14	M5E2	n.a.	BV421	6.0	BioLegend	301829
CD14	M5E2	n.a.	PE	6.0	BioLegend	301805
CD14	MφP9	n.a.	APC	6.0	BD	345787

The fluorochrome-conjugated antibodies were purchased from Agilent Technologies/Dako (Santa Clara, CA, USA), BioLegend (San Diego, CA, USA), BD Biosciences (San Jose, CA, USA), Beckman Coulter (Brea, CA, USA), Tonbo Biosciences (Fremont, CA, USA), Cytek Biosciences (Fremont, CA, USA), and Miltenyi Biotec (Bergisch Gladbach, Germany).

**Table 2:** LAIP/LSC phenotypes identified in frozen patient samples.

Common Primitive Marker	Phenotype	Number of Occurrences (n)	Comment
CD34^+^	CD13^+^CD2^+^	1	
	CD13^+^CD7^+^	4	1 CP
	CD13^+^CD19^+^	1	1 CP
	CD13^+^CD22^+^	2	1 CP
	CD13^+^CD56^+^	3	1 CP
	CD33^+^CD2^+^	1	
	CD33^+^CD7^+^	3	
	CD33^+^CD19^+^	1	
	CD33^+^CD22^+^	1	
	CD33^+^CD56^+^	2	
	CD13^+^HLA-DR^−^	4	
	CD13^−^HLA-DR^+^	7	2 C
	CD13^+^CD33^−^	6	2 C
	CD13^−^CD33^+^	4	1 C
	CD33^+^HLA-DR^−^	1	
	CD13^+^CD11b^+^	3	1 CP
	CD13^+^CD15^+^	6	1 C, 1 CP
	CD33^+^CD11b^+^	2	
	CD33^+^CD15^+^	2	
	CD38^−^CD33^+^	4	LSC
	CD38^−^CD56^+^	1	LSC
	CD38^−^CD45RA^+^	1	LSC
	CD38^−^CD123^+^	6	LSC
CD117^+^	CD13^+^CD7^+^	2	
	CD13^+^CD56^+^	1	
	CD13^+^HLA-DR^−^CD15^−^	3	
	CD13^−^HLA-DR^+^	1	
	CD13^−^CD33^+^	4	1 C
CD133^+^	CD34^−^	5	
	HLA-DR^−^	2	1 C

Summary of all LAIP/LSC phenotypes identified in 18 AML MRD patient samples. Samples were analyzed as fresh specimens using the conventional 5-tube panel on a FACS Canto II and as frozen aliquots using the novel single-tube 22-color panel on the Northern Lights CLC. In total, 26 distinct LAIPs and 4 LSC phenotypes were identified, yielding 84 data points across multiple patients. Abbreviations in the column for comments: C = phenotypes that could not always be identified using the conventional 5-tube assay, because limited cell numbers restricted staining of all tubes and therefore reduced the number of detectable marker combinations. CP = excluded due to cryopreservation-associated differences in LAIP frequencies. The number of excluded LAIPs is indicated; LSC = LSC-associated phenotypes.

## Data Availability

The raw data supporting the conclusions of this article will be made available by the authors on request.

## Supplementary Materials

The following supporting information can be downloaded at: https://www.mdpi.com/article/10.3390/cancers18142323/s1, Table S1: Material used in experiments of this study. Table S2: Average LAIP-frequencies in the KG-1 cell line. Table S3: Preparation of limiting dilution experiments. Table S4: Stain indices for the 22-color panel. Table S5: Determination of LoB, LoD, LoQ for KG-1 derived LAIPs. Table S6: Intra- and inter-assay precision for KG-1 derived LAIPs. Table S7: WBC-MRD on patient samples–spike in experiments. Table S8: WBC-MRD on fresh AML samples. Figure S1: Exemplary Gating Template for Limiting Dilution Experiments. Figure S2: Spectral properties of the 22-color FlowMRD assay. Figure S3: Comparison of nBM cell line unmixing with PB surrogate unmixing. Figure S4: Optimized antibody concentrations reduce data spread and increase data resolution. Figure S5: Accurate unmixing and manual compensation in SpectroFlo. Figure S6: Myeloid differentiation trajectory within the high-dimensional data space and by manual gating. Figure S7: identification of KG-1 cells and KG-1 derived LAIPs within nBM. Figure S8: Hemodilution tracking through CD16 and viability marker assessment. Figure S9: Removed events via PeacoQC. Figure S10: Hemodilution in AML MRD clinical samples.

## References

[1] Lin T.L., Pagano L. (2021). The Important Role of Intensive Induction Chemotherapy in the Treatment of Acute Myeloid Leukemia. Expert Rev. Hematol..

[2] Shimony S., Stahl M., Stone R.M. (2025). Acute Myeloid Leukemia: 2025 Update on Diagnosis, Risk-Stratification, and Management. Am. J. Hematol..

[3] Buccisano F., Hourigan C.S., Walter R.B. (2017). The Prognostic Significance of Measurable (“Minimal”) Residual Disease in Acute Myeloid Leukemia. Curr. Hematol. Malig. Rep..

[4] Short N.J., Zhou S., Fu C., Berry D.A., Walter R.B., Freeman S.D., Hourigan C.S., Huang X., Nogueras Gonzalez G., Hwang H. (2020). Association of Measurable Residual Disease With Survival Outcomes in Patients With Acute Myeloid Leukemia: A Systematic Review and Meta-Analysis. JAMA Oncol..

[5] Heuser M., Freeman S.D., Ossenkoppele G.J., Buccisano F., Hourigan C.S., Ngai L.L., Tettero J.M., Bachas C., Baer C., Béné M.-C. (2021). 2021 Update on MRD in Acute Myeloid Leukemia: A Consensus Document from the European LeukemiaNet MRD Working Party. Blood.

[6] Wood B.L. (2020). Acute Myeloid Leukemia Minimal Residual Disease Detection: The Difference from Normal Approach. Curr. Protoc. Cytom..

[7] Zeijlemaker W., Kelder A., Cloos J., Schuurhuis G.J. (2019). Immunophenotypic Detection of Measurable Residual (Stem Cell) Disease Using LAIP Approach in Acute Myeloid Leukemia. Curr. Protoc. Cytom..

[8] Terwijn M., Kelder A., Snel A.N., Rutten A.P., Scholten W.J., Oussoren Y.J.M., Van De Loosdrecht A.A., Zweegman S., Ossenkoppele G.J., Schuurhuis G.J. (2012). Minimal Residual Disease Detection Defined as the Malignant Fraction of the Total Primitive Stem Cell Compartment Offers Additional Prognostic Information in Acute Myeloid Leukaemia. Int. J. Lab. Hematol..

[9] Hanekamp D., Tettero J.M., Ossenkoppele G.J., Kelder A., Cloos J., Schuurhuis G.J. (2021). AML/Normal Progenitor Balance Instead of Total Tumor Load (MRD) Accounts for Prognostic Impact of Flowcytometric Residual Disease in AML. Cancers.

[10] Li S.-Q., Xu L.-P., Wang Y., Zhang X.-H., Chen H., Chen Y.-H., Wang F.-R., Han W., Sun Y.-Q., Yan C.-H. (2022). An LSC-Based MRD Assay to Complement the Traditional MFC Method for Prediction of AML Relapse: A Prospective Study. Blood.

[11] Zeijlemaker W., Kelder A., Oussoren-Brockhoff Y.J.M., Scholten W.J., Snel A.N., Veldhuizen D., Cloos J., Ossenkoppele G.J., Schuurhuis G.J. (2016). A Simple One-Tube Assay for Immunophenotypical Quantification of Leukemic Stem Cells in Acute Myeloid Leukemia. Leukemia.

[12] Zeijlemaker W., Grob T., Meijer R., Hanekamp D., Kelder A., Carbaat-Ham J.C., Oussoren-Brockhoff Y.J.M., Snel A.N., Veldhuizen D., Scholten W.J. (2019). CD34+CD38− Leukemic Stem Cell Frequency to Predict Outcome in Acute Myeloid Leukemia. Leukemia.

[13] Kersten B., Valkering M., Wouters R., Van Amerongen R., Hanekamp D., Kwidama Z., Valk P., Ossenkoppele G., Zeijlemaker W., Kaspers G. (2016). CD 45 RA, a Specific Marker for Leukaemia Stem Cell Sub-populations in Acute Myeloid Leukaemia. Br. J. Haematol..

[14] Klyuchnikov E., Badbaran A., Massoud R., Freiberger P., Wolschke C., Ayuk F., Fehse B., Bacher U., Kröger N. (2024). Peri-Transplant Flow-MRD Assessment of Cells with Leukemic Stem Cells (LSC) Associated Phenotype in AML Patients Undergoing Allogeneic Stem Cell Transplantation in CR. Leukemia.

[15] Cloos J., Harris J.R., Janssen J.J.W.M., Kelder A., Huang F., Sijm G., Vonk M., Snel A.N., Scheick J.R., Scholten W.J. (2018). Comprehensive Protocol to Sample and Process Bone Marrow for Measuring Measurable Residual Disease and Leukemic Stem Cells in Acute Myeloid Leukemia. J. Vis. Exp..

[16] Soh K.T., Conway A., Liu X., Wallace P.K. (2022). Development of a 27-color Panel for the Detection of Measurable Residual Disease in Patients Diagnosed with Acute Myeloid Leukemia. Cytom. Part A.

[17] Fokken H., Waclawski J., Kattre N., Kloos A., Müller S., Ettinger M., Kacprowski T., Heuser M., Maetzig T., Schwarzer A. (2024). A 19-color Single-tube Full Spectrum Flow Cytometry Assay for the Detection of Measurable Residual Disease in Acute Myeloid Leukemia. Cytom. Part A.

[18] Chen M., Fu M., Gong M., Gao Y., Wang A., Zhao W., Wu X., Wang H. (2023). Twenty-Four-Color Full Spectrum Flow Cytometry Panel for Minimal Residual Disease Detection in Acute Myeloid Leukemia. Open Med..

[19] Zhang Z., Wilhelm M., Sieber I., Döhner H., Feuring M. (2025). Development of a 29-Color Single-Tube Full Spectrum Flow Cytometry Assay for the Detection of Measurable Residual Disease and Leukemic Stem Cells in Acute Myeloid Leukemia. Cytom. Part A.

[20] Nolan J.P. (2022). The Evolution of Spectral Flow Cytometry. Cytom. Part A.

[21] Nolan J.P., Condello D. (2013). Spectral Flow Cytometry. Curr. Protoc. Cytom..

[22] Marsh-Wakefield F.M., Mitchell A.J., Norton S.E., Ashhurst T.M., Leman J.K., Roberts J.M., Harte J.E., McGuire H.M., Kemp R.A. (2021). Making the Most of High-dimensional Cytometry Data. Immunol. Cell Biol..

[23] Den Braanker H., Bongenaar M., Lubberts E. (2021). How to Prepare Spectral Flow Cytometry Datasets for High Dimensional Data Analysis: A Practical Workflow. Front. Immunol..

[24] Shevchenko Y., Lurje I., Tacke F., Hammerich L. (2024). Fluorochrome-dependent Specific Changes in Spectral Profiles Using Different Compensation Beads or Primary Cells in Full Spectrum Cytometry. Cytom. Part A.

[25] Sharma S., Boyer J., Teyton L. (2024). A Practitioner’s View of Spectral Flow Cytometry. Nat. Methods.

[26] Bhowmick D., Lowe S.K., Ratliff M.L. (2023). Side-by-Side Comparison of Compensation Beads Used in Polychromatic Flow Cytometry. ImmunoHorizons.

[27] Park L.M., Lannigan J., Jaimes M.C. (2020). OMIP-069: Forty-Color Full Spectrum Flow Cytometry Panel for Deep Immunophenotyping of Major Cell Subsets in Human Peripheral Blood. Cytom. Part A.

[28] Jensen H.A., Kim J. (2023). iCoreDrop: A Robust Immune Monitoring Spectral Cytometry Assay with Six Open Channels for Biomarker Flexibility. Cytom. Part A.

[29] Loken M.R., Chu S., Fritschle W., Kalnoski M., Wells D.A. (2009). Normalization of Bone Marrow Aspirates for Hemodilution in Flow Cytometric Analyses. Cytom. Part B Clin. Cytom..

[30] Emmaneel A., Quintelier K., Sichien D., Rybakowska P., Marañón C., Alarcón-Riquelme M.E., Van Isterdael G., Van Gassen S., Saeys Y. (2022). PeacoQC: Peak-based Selection of High Quality Cytometry Data. Cytom. Part A.

[31] McInnes L., Healy J., Saul N., Großberger L. (2018). UMAP: Uniform Manifold Approximation and Projection. J. Open Source Softw..

[32] Van Gassen S., Callebaut B., Van Helden M.J., Lambrecht B.N., Demeester P., Dhaene T., Saeys Y. (2015). FlowSOM: Using Self-organizing Maps for Visualization and Interpretation of Cytometry Data. Cytom. Part A.

[33] Delgado J.A., Guillén-Grima F., Moreno C., Panizo C., Pérez-Robles C., Mata J.J., Moreno L., Arana P., Chocarro S., Merino J. (2017). A Simple Flow-Cytometry Method to Evaluate Peripheral Blood Contamination of Bone Marrow Aspirates. J. Immunol. Methods.

[34] Aldawood A.M., Kinkade Z., Rosado F.G., Esan O.A., Gibson L.F., Vos J.A. (2015). A Novel Method to Assess Bone Marrow Purity Is Useful in Determining Blast Percentage by Flow Cytometry in Acute Myeloid Leukemia and Myelodysplasia. Ann. Hematol. Oncol..

[35] Schuurhuis G.J., Heuser M., Freeman S., Béné M.-C., Buccisano F., Cloos J., Grimwade D., Haferlach T., Hills R.K., Hourigan C.S. (2018). Minimal/Measurable Residual Disease in AML: A Consensus Document from the European LeukemiaNet MRD Working Party. Blood.

[36] Hoffmann J., Thrun M.C., Röhnert M.A., Von Bonin M., Oelschlägel U., Neubauer A., Ultsch A., Brendel C. (2023). Identification of Critical Hemodilution by Artificial Intelligence in Bone Marrow Assessed for Minimal Residual Disease Analysis in Acute Myeloid Leukemia: The Cinderella Method. Cytom. Part A.

[37] Flores-Montero J., Sanoja-Flores L., Paiva B., Puig N., García-Sánchez O., Böttcher S., Van Der Velden V.H.J., Pérez-Morán J.-J., Vidriales M.-B., García-Sanz R. (2017). Next Generation Flow for Highly Sensitive and Standardized Detection of Minimal Residual Disease in Multiple Myeloma. Leukemia.

[38] Bardet V., Wagner-Ballon O., Guy J., Morvan C., Debord C., Trimoreau F., Benayoun E., Chapuis N., Freynet N., Rossi C. (2015). Multicentric Study Underlining the Interest of Adding CD5, CD7 and CD56 Expression Assessment to the Flow Cytometric Ogata Score in Myelodysplastic Syndromes and Myelodysplastic/Myeloproliferative Neoplasms. Haematologica.

[39] Van Dongen J.J.M., Lhermitte L., Böttcher S., Almeida J., Van Der Velden V.H.J., Flores-Montero J., Rawstron A., Asnafi V., Lécrevisse Q., on behalf of the EuroFlow Consortium (EU-FP6, LSHB-CT-2006-018708) (2012). EuroFlow Antibody Panels for Standardized N-Dimensional Flow Cytometric Immunophenotyping of Normal, Reactive and Malignant Leukocytes. Leukemia.

[40] Tettero J.M., Dakappagari N., Heidinga M.E., Oussoren-Brockhoff Y., Hanekamp D., Pahuja A., Burns K., Kaur P., Alfonso Z., Van Der Velden V.H.J. (2023). Analytical Assay Validation for Acute Myeloid Leukemia Measurable Residual Disease Assessment by Multiparametric Flow Cytometry. Cytom. Part B Clin. Cytom..

[41] Selliah N., Nash V., Eck S., Green C., Oldaker T., Stewart J., Vitaliti A., Litwin V. (2023). Flow Cytometry Method Validation Protocols. Curr. Protoc..

[42] Tettero J.M., Heidinga M.E., Mocking T.R., Fransen G., Kelder A., Scholten W.J., Snel A.N., Ngai L.L., Bachas C., Van De Loosdrecht A.A. (2024). Impact of Hemodilution on Flow Cytometry Based Measurable Residual Disease Assessment in Acute Myeloid Leukemia. Leukemia.

[43] Armbruster D.A., Pry T. (2008). Limit of Blank, Limit of Detection and Limit of Quantitation. Clin. Biochem. Rev..

[44] Van Dongen J.J.M., Van Der Burg M., Kalina T., Perez-Andres M., Mejstrikova E., Vlkova M., Lopez-Granados E., Wentink M., Kienzler A.-K., Philippé J. (2019). EuroFlow-Based Flowcytometric Diagnostic Screening and Classification of Primary Immunodeficiencies of the Lymphoid System. Front. Immunol..

[45] Wood B., Jevremovic D., Béné M.C., Yan M., Jacobs P., Litwin V., on behalf of ICSH/ICCS Working Group (2013). Validation of Cell-based Fluorescence Assays: Practice Guidelines from the ICSH and ICCS—Part V—Assay Performance Criteria. Cytom. Part B Clin. Cytom..

[46] Van Der Pol M., Pater J., Feller N., Westra A., Van Stijn A., Ossenkoppele G., Broxterman H., Schuurhuis G. (2001). Functional Characterization of Minimal Residual Disease for P-Glycoprotein and Multidrug Resistance Protein Activity in Acute Myeloid Leukemia. Leukemia.

[47] Bill M., Aggerholm A., Kjeldsen E., Roug A.S., Hokland P., Nederby L. (2019). Revisiting CLEC 12A as Leukaemic Stem Cell Marker in AML: Highlighting the Necessity of Precision Diagnostics in Patients Eligible for Targeted Therapy. Br. J. Haematol..

[48] Sakoda T., Kikushige Y., Irifune H., Kawano G., Harada T., Semba Y., Hayashi M., Shima T., Mori Y., Eto T. (2025). TIM-3 Marks Measurable Residual Leukemic Stem Cells Responsible for Relapse after Allogeneic Stem Cell Transplantation. Cancer Sci..

[49] Zhang X., Song B., Carlino M.J., Li G., Ferchen K., Chen M., Thompson E.N., Kain B.N., Schnell D., Thakkar K. (2024). An Immunophenotype-Coupled Transcriptomic Atlas of Human Hematopoietic Progenitors. Nat. Immunol..

[50] Lokwani R., Chaudhari R., Wolf M.T., Sadtler K. (2022). Spectral Cytometry on Highly Autofluorescent Samples. Nat. Rev. Methods Primer.

[51] Nguyen R., Perfetto S., Mahnke Y.D., Chattopadhyay P., Roederer M. (2013). Quantifying Spillover Spreading for Comparing Instrument Performance and Aiding in Multicolor Panel Design. Cytom. Part A.

[52] Parks D.R. (2020). Multispectral Flow Cytometry: Unaddressed Issues and Recommendations for Improvement. Cytom. Part A.

[53] Roca C.P., Burton O.T., Gergelits V., Prezzemolo T., Whyte C.E., Halpert R., Kreft Ł., Collier J., Botzki A., Spidlen J. (2021). AutoSpill Is a Principled Framework That Simplifies the Analysis of Multichromatic Flow Cytometry Data. Nat. Commun..

[54] Terwijn M., Zeijlemaker W., Kelder A., Rutten A.P., Snel A.N., Scholten W.J., Pabst T., Verhoef G., Löwenberg B., Zweegman S. (2014). Leukemic Stem Cell Frequency: A Strong Biomarker for Clinical Outcome in Acute Myeloid Leukemia. PLoS ONE.

[55] Mage P.L., Konecny A.J., Mair F. (2026). Measurement and Prediction of Unmixing-Dependent Spreading Due to Collinearity in Spectral Flow Cytometry. Cytom. Part A.

[56] Ferrer-Font L., Pellefigues C., Mayer J.U., Small S.J., Jaimes M.C., Price K.M. (2020). Panel Design and Optimization for High-Dimensional Immunophenotyping Assays Using Spectral Flow Cytometry. Curr. Protoc. Cytom..

[57] McCausland M., Lin Y.-D., Nevers T., Groves C., Decman V. (2021). With Great Power Comes Great Responsibility: High-Dimensional Spectral Flow Cytometry to Support Clinical Trials. Bioanalysis.

