# Supplementary material for: A Clinically Applicable Unmixing Approach for Spectral MRD Detection in AML
Source: Cancers (Basel). 2026 Jul 18;18(14):2323. doi: 10.3390/cancers18142323 (PMC13406482; doi:10.3390/cancers18142323)
Supplement: Supplementary file 1 [file cancers-18-02323-s001.zip › cancers-4385216-supplementary.pdf]

Supplementary Material

Table S1: Materials used in experiments of this study

**Material**	**Total amount used**
KG-1 cell-line	1
nBM	17
PB	5-10 pooled per measurement of single stained references
AML diagnosis samples	3
AML MRD samples	23
➔ frozen/thawed	18
➔ fresh	5

**Validation experiment**	**Material used**
Hemodilution spike-in experiments	1xnBM, 1xPB, 1xKG-1 cell-line
LAIP spike-in experiments	1xnBM, 1xKG-1 cell-line
spike-in experiments of AML diagnosis samples in nBM	4xnBM, 3xAML diagnosis samples

Table S2: Average LAIP-frequencies in the KG-1 cell-line

The KG-1 cell-line contains the LAIPs: CD34$^+$CD13$^+$CD7$^+$, CD34$^+$CD13$^+$CD56$^+$, CD34$^+$CD13$^+$CD33$^-$, CD34$^+$CD13$^+$HLA-DR$^-$. Mean LAIP-contents have been determined via manual gating of KG-1 cell data in Infinicyt. The LAIP frequency was determined in two independent measurements.

	**CD34$^+$CD13$^+$CD7$^+$**	**CD34$^+$CD13$^+$CD56$^+$**	**CD34$^+$CD13$^+$CD33$^-$**	**CD34$^+$CD13$^+$HLA-DR$^-$**
**Mean LAIP-content [%]**	83.48	64.76	82.56	79.49

Table S3: Preparation of limiting dilution experiments

The following table gives an overview of the analyzed performance characteristics and used dilution steps of the different validation experiments (intra-/inter-assay precision (coefficient of variation - CV) and measurement of limits of blank (LoB), detection (LoD) and quantitation (LoQ)) – n.a. stands for not applicable.

	**Performance characteristic**			**Dilutions**
	**Intra-assay precision**	**Inter-assay precision**	**Limit of blank, detection and quantitation (LoB, LoD, LoQ)**	
**Hemodilution**	determined	n.a.	n.a.	KG1-nBM-dilution:<br>0%, 0.3%, 100%<br>PB-BM-dilution:<br>0%, 10%, 20%, 40%, 100%
**LAIP**	determined	determined	determined	KG1-nBM-dilution:<br>0%, 0.005%, 0.010%, 0.015%, 0.025%, 0.05%, 0.1%, 0.5%, 100%

Table S4: Stain indices for the 22-color panel

Rows that are marked blue were copied from the parental 19-color panel from Fokken et al. 2024 (https://pubmed.ncbi.nlm.nih.gov/37984809/). In the 22-color panel these markers were unchanged in comparison to the 19-color panel and therefore we assume the same stain indices, as well as use the same antibody amounts for these marker fluorochrome combinations.

For the calculation of the stain indices (SI) the following formula was used: $SI = \frac{MFI^{+} - MFI^{-}}{2 \cdot SD^{-}}$, in which $MFI^{+/-}$ stands for the mean fluorescent intensity of the marker positive/negative population respectively and SD^{-} stands for the standard deviation of the negative population. These values were determined for each marker via manual gating in Infinicyt, performed on single stains with the dilution steps of the antibodies mentioned in the tables below.

The final antibody amounts were chosen based upon the optimal stain index and an estimation of the unmixing results regarding spillover and spread between different fluorochrome channels (Figure S4).

Marker	Clone	Fluorochrome	Stain Index			
			1:25	1:100	1:400	1:1600
CD2	MT910	FITC	11.66	12.59	10.86	5.26
CD4	SK3	Spark Blue 550	33.55	40.84	38.97	15.05
CD7	M-T701	BV786	32.46	45.52	20.34	16.99
CD11b	D12	BV750	9.72	11.45	12.00	9.17
CD13	WM15	BV785	7.22	7.29	9.23	3.09
CD14	MϕP9	BV650	25.15	24.83	14.69	3.68
CD15	MMA	Pacific Blue	23.37	28.34	20.72	7.67
CD16	3G8	BV570	4.91	4.28	3.88	3.25
CD19	SJ25C1	PE-Cy5	28.99	35.03	23.16	16.27
CD22	S-HCL1	APC-Fire 750	14.48	7.95	4.58	9.01
CD33	P67.6	APC-R700	61.04	33.26	16.96	5.72
CD34	8G12	BV421	68.72	53.41	33.58	9.95
CD38	HB7	APC-Fire 810	21.22	21.50	21.27	16.81
CD45	J.33	Krome Orange	123.91	76.79	36.08	16.53
CD45RA	HI100	SparkNIR685	35.62	37.72	15.99	10.17
CD56	MY31	PE-Cy7	6.21	8.67	5.24	3.91
CD64	10.1	BV605	2.75	2.16	0.81	-0.09
CD123	7G3	BV480	4.81	6.11	5.78	5.20
HLA-DR	L243	PE-Fire 810	57.92	50.50	25.39	16.26

Marker	Clone	Fluorochrome	Stain Index			
			1:5	1:20	1:80	1:320
CD117	104D2D1	PE	3.46	6.58	6.69	4.04

Marker	Clone	Fluorochrome	Stain Index			
			1:50	1:200	1:800	1:3200
CD133	AC133	APC	79.53	57.56	24.89	17.54

Marker	Clone	Fluorochrome	Stain Index			
			1:100	1:400	1:1000	1:1600
viability	/	Zombie NIR	141.31	69.04	81.42	62.80

Table S5: Determination of LoB, LoD and LoQ for KG1-derived LAIPs

Limit of blank, detection and quantitation (LoB, LoD, LoQ) for conventional LAIPs:

- ➔ The mean and standard-deviation (SD) of the LAIP-content were calculated in the dilutions of nBM with KG-1 cells, as well as in the pure nBM.
- ➔ The coefficient of variation (CV) was calculated: CV = SD / mean LAIP-content.
- ➔ LoB = mean (nBM LAIP-content) + 1.645*SD (nBM LAIP-content)
- ➔ LoD = mean (nBM LAIP-content) + 3.29*SD (nBM LAIP-content)
- ➔ LoQ = mean LAIP-content of the dilution step, which suffices the following two conditions:
  1. The mean LAIP-content is strictly higher than the LoD (> LoD),
  2. The coefficient of variation (CV) is strictly lower than 25% (CV<25%).

	LoQ	LoD	LoB
**$CD34^+CD13^+CD7^+$**	0.00778 %	0.00219 %	0.00167%
**$CD34^+CD13^+CD56^+$**	0.00693 %	0.00147 %	0.00127 %
**$CD34^+CD13^+CD33^-$**	0.00512 %	0.00506 %	0.00324 %
**$CD34^+CD13^+HLA\text{-}DR^-$**	0.00639 %	0.00757 %	0.00551 %

Acceptance criterion:
For conventional LAIPs the LoQ should be beneath 0.01 % (MRD-positivity cut-off of 0.1%), which all conventional LAIPs of the KG1-cell-line passed.

Table S6: Intra- and inter-assay precision for KG1-derived LAIPs:

Intra- and inter-assay precision were measured by the coefficient of variation (CV). The CV-value gets calculated via the standard deviation (SD) and mean LAIP-content of a triplicate via the following formula: CV = SD / mean LAIP-content. In the following tables, the CVs in [%] for intra- and inter-assay precision experiments are listed.

LAIP dilution experiments: Intra- and inter-assay precision

$CD34^+CD13^+CD7^+$ Dilution step [%]	Intra-Assay Run 1	Intra-Assay Run 2	Inter-Assay
**0.5**	2.38	3.13	3.82
**0.1**	8.37	6.29	7.79
**0.05**	4.78	3.98	3.95
**0.025**	11.16	/	/
**0.015**	17.48	/	/
**0.010**	7.77	/	/
**0.005**	18.83	/	/
**Mean CV [%]**	10.11	4.47	5.19

$CD34^+CD13^+CD56^+$ Dilution step [%]	Intra-Assay Run 1	Intra-Assay Run 2	Inter-Assay
**0.5**	1.52	4.90	4.11
**0.1**	5.91	6.10	7.73
**0.05**	2.93	0.88	1.95
**0.025**	7.83	/	/
**0.015**	13.45	/	/
**0.010**	2.48	/	/
**0.005**	18.04	/	/
**Mean CV [%]**	7.45	3.96	4.60

$CD34^+CD13^+CD33^-$ Dilution step [%]	Intra-Assay Run 1	Intra-Assay Run 2	Inter-Assay
**0.5**	3.26	3.90	3.94
**0.1**	6.90	6.00	7.94
**0.05**	6.30	0.71	4.62
**0.025**	11.82	/	/
**0.015**	16.34	/	/
**0.010**	8.50	/	/
**0.005**	8.06	/	/
**Mean CV [%]**	8.74	3.54	5.50

$CD34^+CD13^+HLA\text{-}DR^-$ Dilution step [%]	Intra-Assay Run 1	Intra-Assay Run 2	Inter-Assay
**0.5**	0.78	1.61	2.86
**0.1**	10.35	5.72	8.62
**0.05**	8.24	4.69	7.31
**0.025**	14.80	/	/
**0.015**	9.84	/	/
**0.010**	15.16	/	/
**0.005**	2.04	/	/
**Mean CV [%]**	8.75	4.01	6.26

Hemodilution experiments:

WBC-MRD: Intra-assay-precision

Dilution Step [%]	$CD34^+CD13^+CD7^+$	$CD34^+CD13^+CD56^+$	$CD34^+CD13^+CD33^-$	$CD34^+CD13^+HLA\text{-}DR^-$
**0**	5.90	4.77	4.94	4.22
**10**	2.82	18.10	3.89	5.36
**20**	5.25	18.02	3.05	4.35
**40**	8.86	8.92	9.47	10.08
**Mean CV [%]**	5.71	12.45	5.34	6.00

The CV was calculated for each PB dilution step and the mean CV was defined by taking the mean of all single CV values from the respective dilution steps.

PM-MRD: Intra-assay-precision

	$CD34^+CD13^+CD7^+$	$CD34^+CD13^+CD56^+$	$CD34^+CD13^+CD33^-$	$CD34^+CD13^+HLA\text{-}DR^-$
**Mean CV [%]**	10.57	9.92	10.45	11.31

As PM-MRD is stable under hemodilution (Tettero et al.; doi: 10.1038/s41375-024-02158-1), the CV was calculated through taking the standard deviation and mean PM-MRD LAIP-content throughout all PB dilution steps (0 - 40 %).

Acceptance criteria:
The CV for intra- and inter-assay precision was defined as acceptable beneath 25% and desired below 10%. All CVs met the acceptance criterion (CV< 20%), whilst most of the data also passed the desired cut-off (CV<10%).

Table S7: WBC MRD on patient samples – spike in experiments

This table shows the LAIP frequencies in percentages of the total WBC compartment for all LAIPs found in three distinct AML diagnosis samples spiked into normal bone marrow, respectively in dilution steps of 1:10 – 1:1250. The LAIPs $CD117^+CD13^+CD7^+$ and $CD117^+CD13^+HLADR^-$ appeared in two distinct patient samples, which is why there are separated rows for these two LAIPs.

**Dilution**	**1:10**		**1:50**		**1:250**		**1:1250**	
**Machine**	**NL**	**Canto**	**NL**	**Canto**	**NL**	**Canto**	**NL**	**Canto**
**$CD34^+CD13^+CD7^+$**	0.890	1.226	0.217	0.324	0.055	0.087	0.017	0.041
**$CD34^+CD13^+HLADR^-$**	0.251	0.256	0.058	0.065	0.013	0.020	0.004	0.007
**$CD117^+CD13^+CD7^+$**	2.953	3.388	0.678	0.930	0.166	0.221	0.041	0.077
	0.373	0.205	0.072	0.062	0.016	0.024	0.014	0.032
**$CD117^+CD13^-CD33^+$**	9.894	10.329	2.198	2.433	0.494	0.566	0.206	0.204
**$CD117^+CD13^+CD33^-$**	0.512	0.223	0.142	0.065	0.078	0.029	0.080	0.021
**$CD117^+CD13^+HLADR^-$**	1.310	1.058	0.291	0.267	0.071	0.068	0.016	0.017
	0.761	0.342	0.145	0.087	0.034	0.027	0.019	0.017
**$CD133^+CD34^-$**	1.276	1.199	0.312	0.305	0.094	0.084	0.043	0.030

This table shows the relative fold differences between the LAIP analysis in the NL measurements (22-color panel) and the corresponding Canto measurement (5 tube panel), represented by the quotient of the NL LAIP size to the Canto LAIP size (NL/Canto). Relative coefficients >2 are marked as red.

**LAIP**	**1:10**	**1:50**	**1:250**	**1:1250**
**$CD34^+CD13^+CD7^+$**	0.726	0.670	0.632	0.415
**$CD34^+CD13^+HLADR^-$**	0.980	0.892	0.650	0.571
**$CD117^+CD13^+CD7^+$**	0.872	0.729	0.751	0.532
	1.820	1.161	0.667	0.438
**$CD117^+CD13^-CD33^+$**	0.958	0.903	0.873	1.010
**$CD117^+CD13^+CD33^-$**	2.296	2.185	2.690	3.810
**$CD117^+CD13^+HLADR^-$**	1.238	1.090	1.044	0.941
	2.225	1.667	1.259	1.118
**$CD133^+CD34^-$**	1.064	1.023	1.119	1.433

Table S8: WBC MRD on fresh AML samples

This table shows the LAIP frequencies in percentages of the total WBC compartment for all LAIPs found in five distinct fresh AML MRD patient samples. The $CD34^+CD13^-HLA\text{-}DR^+$ LAIP appeared in two distinct patient samples, which is why there are separated rows for this LAIP.

**LAIP**	**NL**	**Canto**
**$CD117^+CD13^-HLA\text{-}DR^+$**	0.171	0.187
**$CD34^+CD13^-HLA\text{-}DR^+$**	0.137	0.150
	0.586	0.630
**$CD117^+CD13^-CD33^+$**	2.087	2.247
**$CD133^+CD34^-$**	0.700	0.640

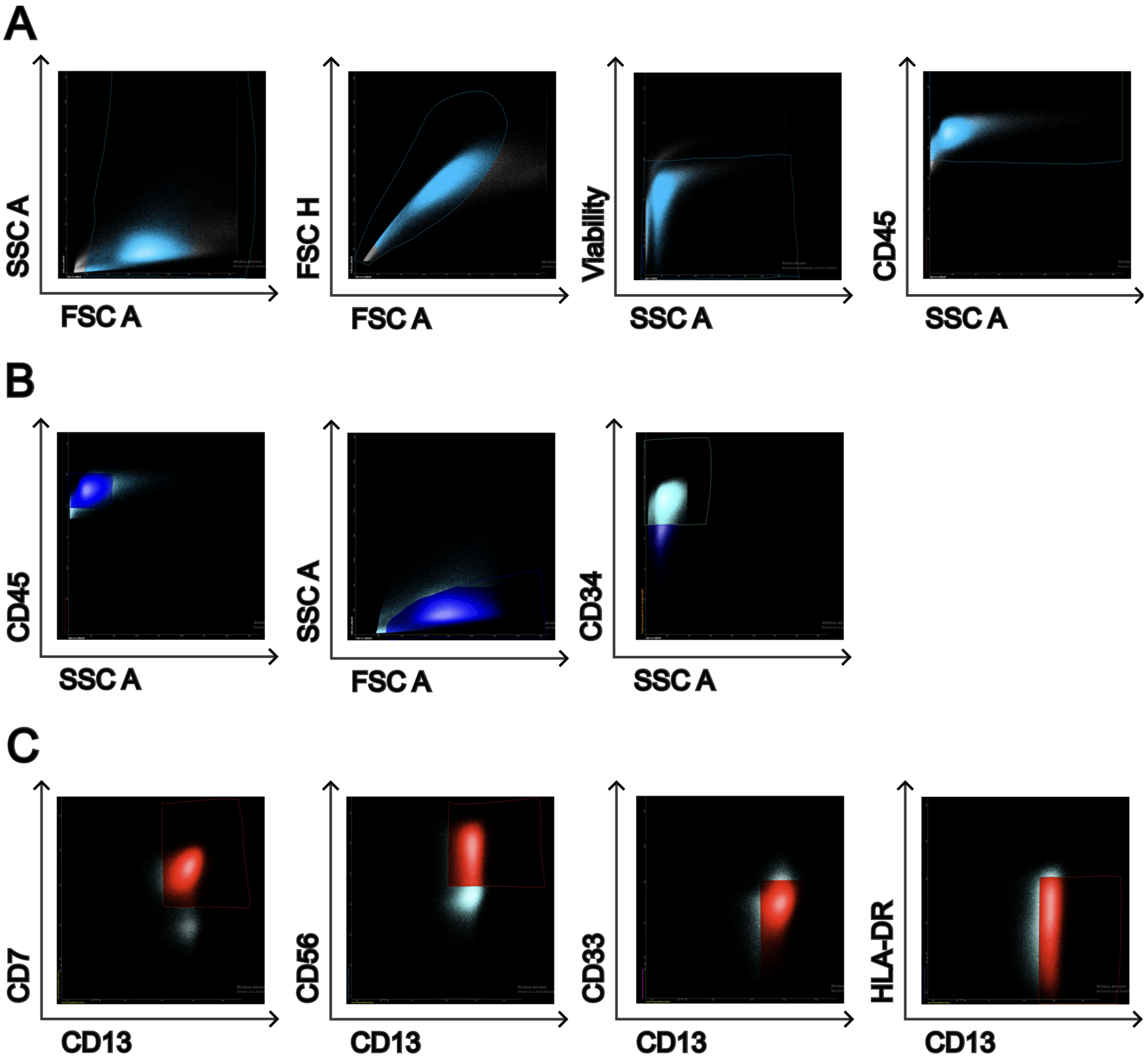

Figure S1: Exemplary gating template for limiting dilution experiments

The KG-1 cell-line was stained with the 22-color panel and was measured on the Cytek NL flow cytometer. Unmixing through the PB surrogate approach and a manual analysis of the KG-1 cell-line in Infinicyt was performed and saved as a gating template. This was used for the analysis of the limiting dilution experiment for the LAIPs of the KG-1 cell-line. An analogous template was created for the hemodilution spike-in experiment. **(A)** Gating of WBCs via exclusion of debry, doublets, dead cells and $CD45^-$ cells. **(B)** Gating of KG-1 blasts via scatter characteristics and $CD34^+$ cells. **(C)** Gating of the characteristic KG-1 LAIP populations in the $CD34^+$ blast population: $CD7^+CD13^+$, $CD56^+CD13^+$, $CD33^-CD13^+$, $HLADR^-CD13^+$.

A

	BV421	Pacific Blue	BV480	Krome Orange	BV570	BV605	BV650	BV750	BV785	FITC	Spark Blue 550	PE	PE-CF594	PE-Cy5	PE-Cy7	PE-Fire 810	APC	Spark NIR 685	APC-R 700	Zombie NIR	APC-Fire 750	APC-Fire 810
BV421	1.00																					
Pacific Blue	0.75	1.00																				
BV480	0.28	0.59	1.00																			
Krome Orange	0.09	0.22	0.70	1.00																		
BV570	0.20	0.18	0.28	0.66	1.00																	
BV605	0.12	0.12	0.22	0.50	0.71	1.00																
BV650	0.18	0.15	0.12	0.19	0.30	0.58	1.00															
BV750	0.06	0.05	0.03	0.04	0.05	0.12	0.24	1.00														
BV785	0.13	0.10	0.04	0.03	0.05	0.09	0.17	0.81	1.00													
FITC	0.03	0.04	0.12	0.07	0.05	0.06	0.04	0.02	0.01	1.00												
Spark Blue 550	0.02	0.04	0.17	0.25	0.20	0.16	0.09	0.02	0.02	0.64	1.00											
PE	0.02	0.03	0.12	0.29	0.52	0.28	0.11	0.02	0.01	0.13	0.41	1.00										
PE-CF594	0.01	0.01	0.05	0.13	0.25	0.37	0.21	0.04	0.03	0.06	0.25	0.43	1.00									
PE-Cy5	0.00	0.00	0.01	0.02	0.05	0.11	0.29	0.07	0.04	0.02	0.10	0.11	0.48	1.00								
PE-Cy7	0.01	0.00	0.00	0.01	0.01	0.02	0.04	0.21	0.23	0.01	0.03	0.03	0.07	0.13	1.00							
PE-Fire 810	0.01	0.01	0.02	0.03	0.06	0.04	0.03	0.10	0.13	0.03	0.09	0.17	0.12	0.11	0.75	1.00						
APC	0.01	0.01	0.01	0.02	0.03	0.07	0.41	0.07	0.04	0.01	0.01	0.01	0.03	0.40	0.02	0.01	1.00					
Spark NIR 685	0.00	0.00	0.00	0.01	0.01	0.02	0.27	0.07	0.04	0.00	0.00	0.00	0.01	0.35	0.03	0.01	0.72	1.00				
APC-R 700	0.03	0.03	0.03	0.03	0.03	0.04	0.23	0.13	0.08	0.02	0.02	0.02	0.03	0.26	0.05	0.02	0.51	0.79	1.00			
Zombie NIR	0.01	0.01	0.01	0.01	0.01	0.03	0.12	0.33	0.27	0.01	0.01	0.01	0.03	0.17	0.33	0.17	0.27	0.40	0.57	1.00		
APC-Fire 750	0.01	0.00	0.00	0.00	0.00	0.01	0.06	0.19	0.24	0.00	0.00	0.00	0.00	0.07	0.13	0.06	0.16	0.26	0.32	0.74	1.00	
APC-Fire 810	0.01	0.01	0.00	0.00	0.01	0.01	0.07	0.13	0.21	0.00	0.00	0.00	0.01	0.07	0.10	0.10	0.16	0.20	0.23	0.41	0.76	1.00

Complexity Index 8.19

B

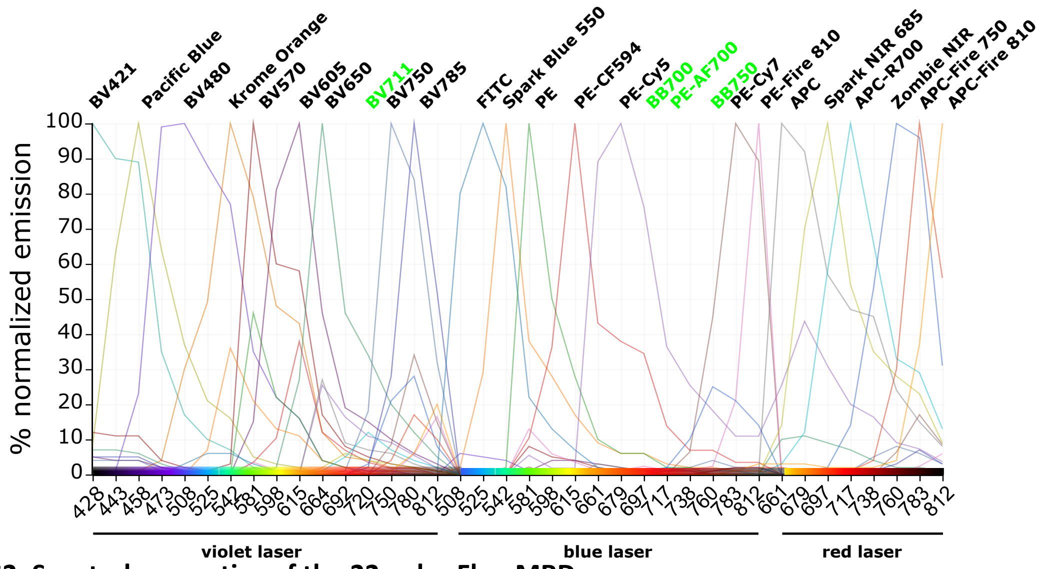

Figure S2: Spectral properties of the 22-color FlowMRD assay.
(**A**) Similarity matrix and complexity index extracted from the SpectroFlo software (Cytek Biosciences). Higher values indicate higher spectral similarities. (**B**) Normalized emmisson spectra extracted from the Cytek Spectral Viewer. Green flurochromes represent drop-in opportunities.

A

BM:

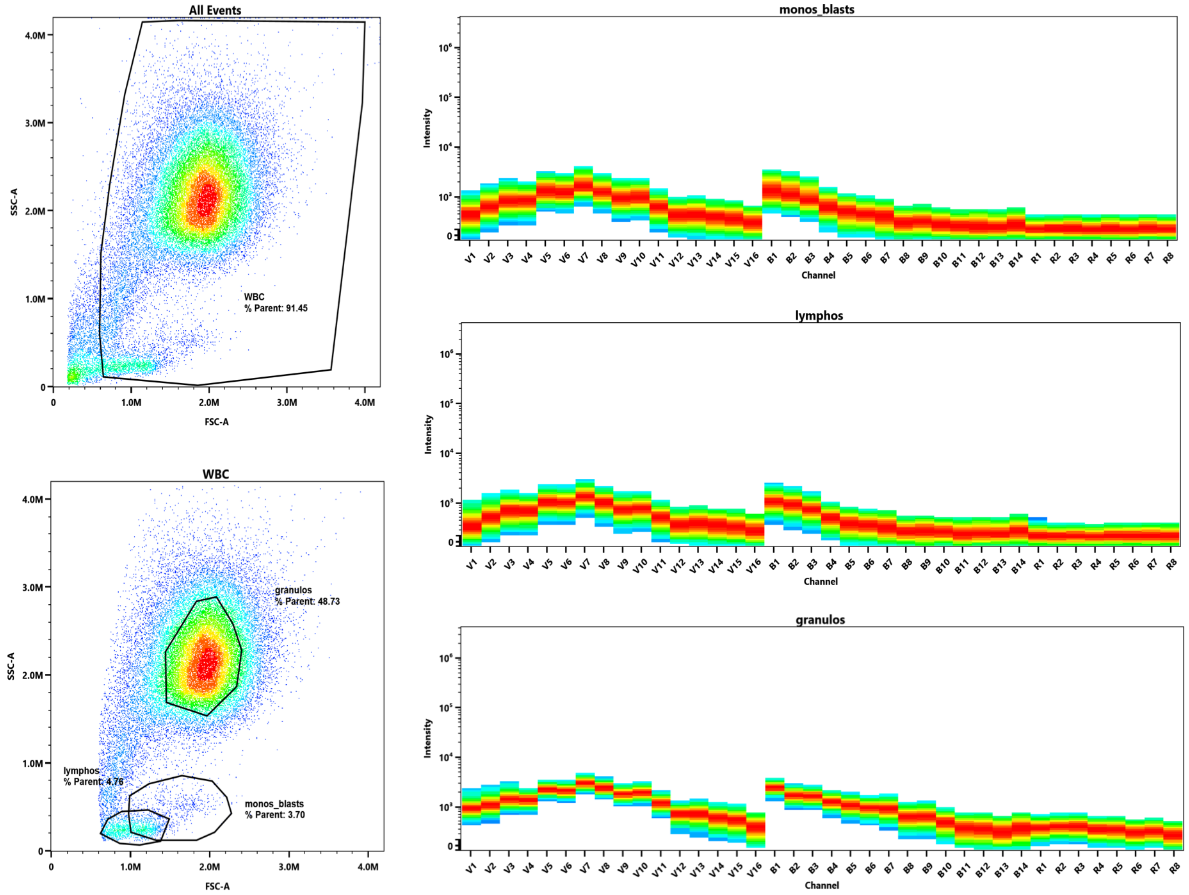
All Events
SSC-A
FSC-A
0
1.0M
2.0M
3.0M
4.0M
WBC
% Parent: 91.45
WBC
granulos
% Parent: 48.73
lymphos
% Parent: 4.76
monos_blasts
% Parent: 3.70
monos_blasts
lymphos
granulos
Intensity
Channel
V1 V2 V3 V4 V5 V6 V7 V8 V9 V10 V11 V12 V13 V14 V15 V16 B1 B2 B3 B4 B5 B6 B7 B8 B9 B10 B11 B12 B13 B14 R1 R2 R3 R4 R5 R6 R7 R8

PB:

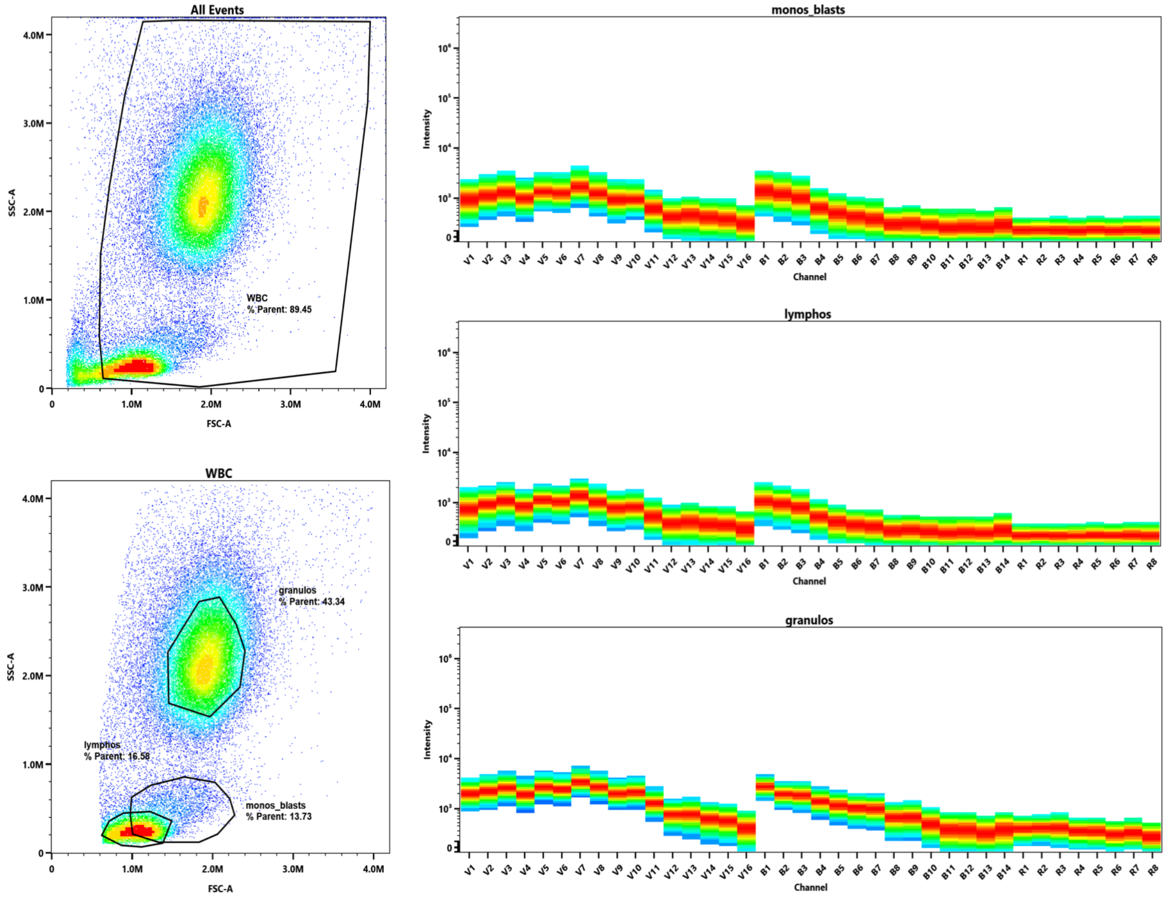
All Events
SSC-A
FSC-A
0
1.0M
2.0M
3.0M
4.0M
WBC
% Parent: 89.45
WBC
granulos
% Parent: 43.34
lymphos
% Parent: 16.58
monos_blasts
% Parent: 13.73
monos_blasts
lymphos
granulos
Intensity
Channel
V1 V2 V3 V4 V5 V6 V7 V8 V9 V10 V11 V12 V13 V14 V15 V16 B1 B2 B3 B4 B5 B6 B7 B8 B9 B10 B11 B12 B13 B14 R1 R2 R3 R4 R5 R6 R7 R8

B

BM:

INTO ↓ FROM →	BV421	Pacific Blue	BV480	Krome Orange	BV570	BV605	BV650	BV750	BV785	FITC	cFluor B548	PE	PE-CF594	PE-Cy5	RB780	PE Fire 810	APC	Spark NIR 685	APC-R700	Zombie NIR	APC-Fire 750	APC-Fire 810	AF
BV421	100	0	0	0	-1.45	0	0	0	-1.15	0	0	0	0	0	0	0	0	-1.65	0	0	0	0	0
Pacific Blue	0	100	0	0	0	0	0	-0.53	0	0	0	0	0	0	0	0	0	0	0	0	0	0	0
BV480	0	0	100	0	0	0	0	0	0	0	0	0	0	0	0	0	0	0	0	0	0	0	0
Krome Orange	0	0	0	100	0	0	0	0	0	0	0	0	0	0	0	0	0	0	0	0	0	0	0
BV570	0	0	0	0	100	0	0	0	0	0	0	0	0	0	0	0	0	0	0	0	0	0	0
BV605	0	0	0	0	0	100	0	0	0	0	0	0	0	0	0	0	0	0	0	0	0	0	0
BV650	0	0	0	0	0	0	100	0	0	0	0	0	0	0	0	0	0	0	0	0	0	0	0
BV750	0	0	0	0	0	0	0	100	0	0	0	0	0	0	0	0	0	0	0	0	0	0	0
BV785	0	0	0	0	0	0	0	0	100	0	0	0	0	0	0	-0.63	0	0	0	0	0	0	0
FITC	0	0	0	0	0	0	0	0	0	100	0	0	0	0	0	0	0	0	0	0	0	0	0
cFluor B548	0	0	0	0	-0.63	0	0	0	0	0	100	0	0	0	0	0	0	0	0	0	0	0	0
PE	0	0	0	0	-0.73	0	0	0	0	0	0	100	0	0	0	-0.46	0	0	0	0	0	0	0
PE-CF594	0	0	0	0	0	0	0	0	0	0	0	0	100	0	0	0.32	0	0	0	0	0	0	0
PE-Cy5	0	0	0	0	0	0	0	0	0	0	0	0	-0.54	100	0	0	0	0	0	0	0	0	0
RB780	0	0	0	0	0	0	0	0	0	0	0	0	0	0	100	0	0	0	0	-2.99	0	0	0
PE Fire 810	0	0	0	0	-1.81	0	0	0	0	0	0	0	0	0	0	100	0	0	0	0	0	0	0
APC	0	0	0	-1	0	0	0	0	0	0	0	0	0	-2.07	0	0	100	0	0	0	0	0	0
Spark NIR 685	0	0	0	0	0	0	0	0	0	0	0	0	0	0	0	0	0	100	-2.86	0	0	0	0
APC-R700	0	0	0	0	0	0	0	0	0	0	0	0	0	0	0	0	0	0	100	0	0	0	0
Zombie NIR	0	0	0	0	0	0	0	1.17	0	0	0	0	0	0	0	0	0	0	0	100	0	0	0
APC-Fire 750	0	0	0	0	0	0	0	0	0	0	0	0	0	0	0	0	0	0	0	0	100	0	0
APC-Fire 810	0	0	0	0	0	0	0	-0.01	0	0	0	0	0	0	0	0	0	0	0	0	0	100	0
AF	0	0	0	0	0	0	0	0	0	0	0	0	0	0	0	0	0	0	0	0	0	0	100

PB:

INTO ↓ FROM →	BV421	Pacific Blue	BV480	Krome Orange	BV570	BV605	BV650	BV750	BV785	FITC	cFluor B548	PE	PE-CF594	PE-Cy5	RB780	PE Fire 810	APC	Spark NIR 685	APC-R700	Zombie NIR	APC-Fire 750	APC-Fire 810	AF
BV421	100	0	0	0	-1.45	0	0	-0.57	-1.15	-2.5	0	0	0	0	0	0	0	-1.65	0	0	0	0	0
Pacific Blue	0	100	-2.04	0	0	0	0	-0.53	0	0	0	0	0	0	0	0	0	0	0	0	0	0	0
BV480	0	0	100	0	0	0	0	0	0	0	0	0	0	0	0	0	0	0	0	0	0	0	0
Krome Orange	0	0	0	100	0	0	0	0	0	0	0	0	0	0	0	0	0	0	0	0	0	0	0
BV570	0	0	0	0	100	0	0	0	0	0	0	0	0	0	0	0	0	0	0	0	0	0	0
BV605	0	0	0	0	0	100	0	0	0	0	0	0	0	0	0	0	0	0	0	0	0	0	0
BV650	0	0	0	0	0	0	100	0	0	0	0	0	0	0	0	0	0	0	0	0	0	0	0
BV750	0	0	0	0	0	0	0	100	0	0	0	0	0	0	0	0	0	0	0	0	0	0	0
BV785	0	0	0	0	0	0	0	0	100	0	0	0	0	0	0	-0.59	0	0	0	0	0	0	0
FITC	0	0	0	0	0	0	0	-0.36	0	100	0	0	0	0	0	0	0	0	0	0	0	0	0
cFluor B548	0	0	0	0	-0.63	0	0	0	0	-1.56	100	0	0	0	0	0	0	0	0	0	0	0	0
PE	0	0	0	0	-0.73	0	0	0	0	0	0	100	0	0	0	-0.82	0	0	0	0	0	0	0
PE-CF594	0	0	0	0	0	0	0	0	0	0	0	0	100	0	0	0.17	0	0	0	0	0	0	0
PE-Cy5	0	0	0	0	0	0	0	0	0	0	0	0	-0.02	100	0	0	0	0	0	0	0	0	0
RB780	0	0	0	0	0	0	0	0	0	0	0	0	0	0	100	0	0	0	0	-2.99	0	0	0
PE Fire 810	0	0	0	0	-1.81	0	0	0	0	0	0	0	0	0	0	100	0	0	0	0	0	0	0
APC	0	0	0	-1	0	0	-0.97	-0.32	0	0	0	0	0	-1.54	0	0	100	0	0	0	0	-0.84	0
Spark NIR 685	0	0	0	0	0	0	0	0	0	0	0	0	0	0	0	0	0	100	-3	0	0	0	0
APC-R700	0	0	0	0	0	0	0	0	0	0	0	0	0	0	0	0	0	0	100	0	0	0	0
Zombie NIR	0	0	0	0	0	0	0	1.17	0	0	0	0	0	0	0	0	0	0	0	100	0	0	0
APC-Fire 750	0	0	0	0	0	0	0	0	0	0	0	0	0	0	0	0	0	0	0	0	100	0	0
APC-Fire 810	0	0	0	0	0	0	0	-0.01	0	0	0	0	0	0	0	0	0	0	0	0	0	100	0
AF	0	0	0	0	0	0	0	0	0	0	0	0	0	0	0	0	0	0	0	0	0	0	100

C

BM:

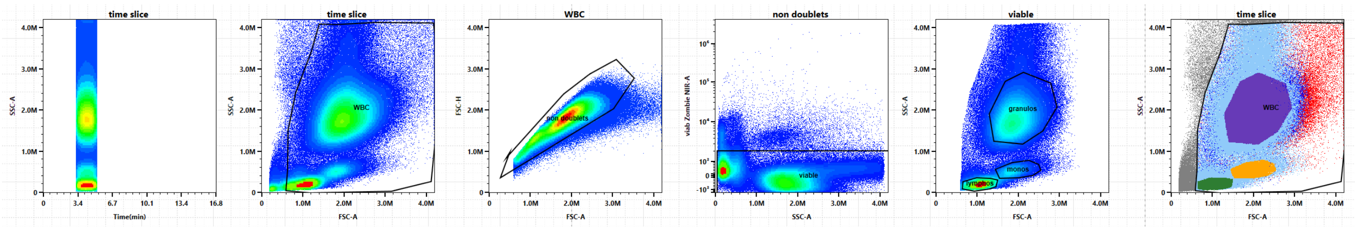

PB:

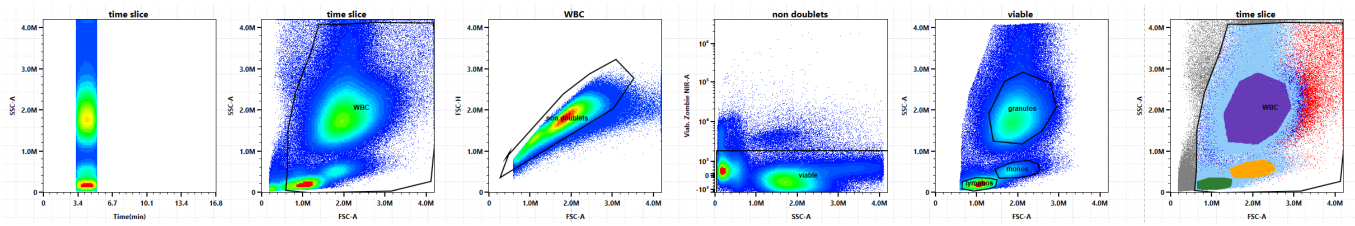

D

CD4

BM:

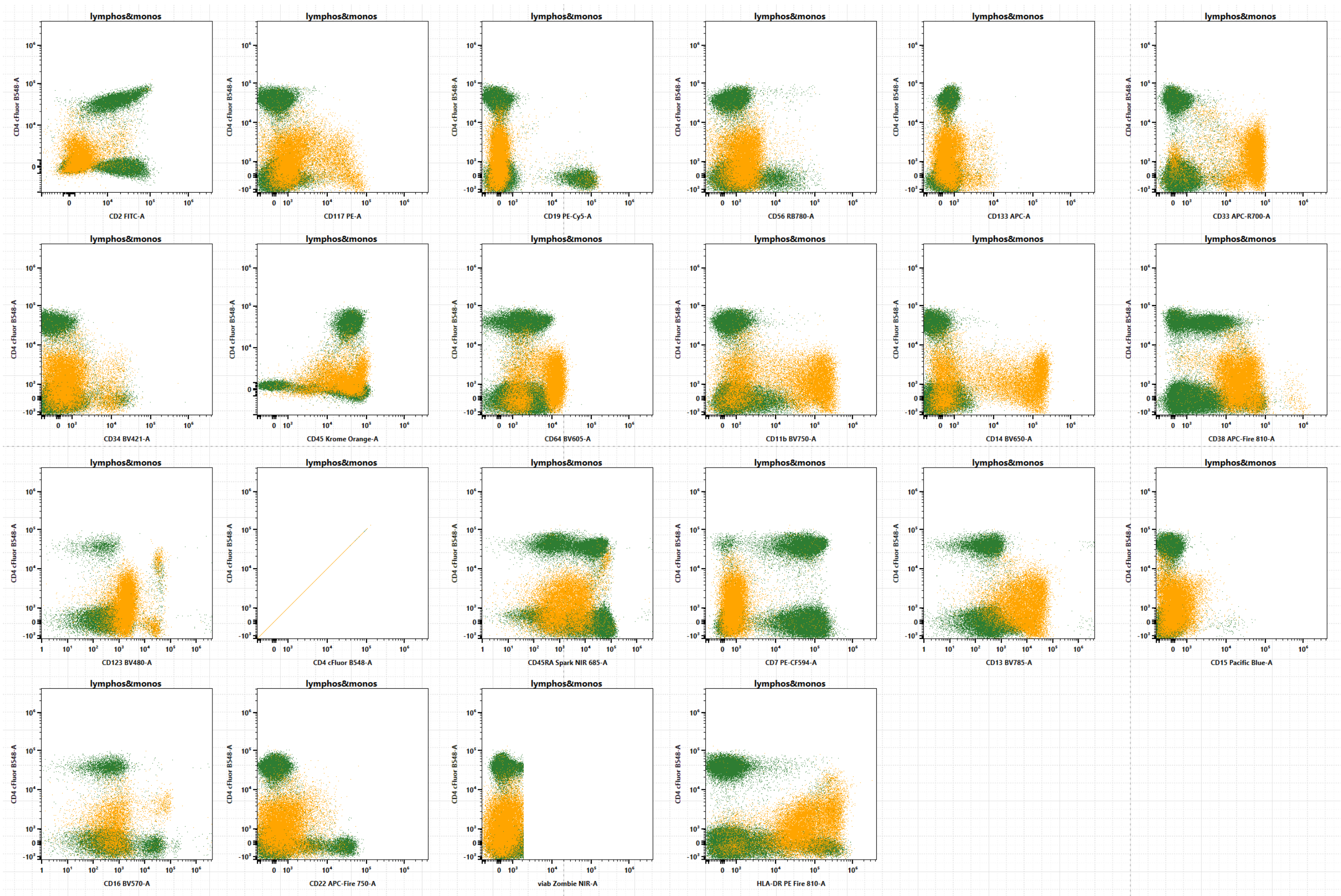
lymphos&monos
CD4 cFluor B548-A
CD2 FITC-A
CD117 PE-A
CD19 PE-Cy5-A
CD56 RB780-A
CD133 APC-A
CD33 APC-R700-A
CD34 BV421-A
CD45 Krome Orange-A
CD64 BV605-A
CD11b BV750-A
CD14 BV650-A
CD38 APC-Fire 810-A
CD123 BV480-A
CD4 cFluor B548-A
CD45RA Spark NIR 685-A
CD7 PE-CF594-A
CD13 BV785-A
CD15 Pacific Blue-A
CD16 BV570-A
CD22 APC-Fire 750-A
viab Zombie NIR-A
HLA-DR PE Fire 810-A

PB:

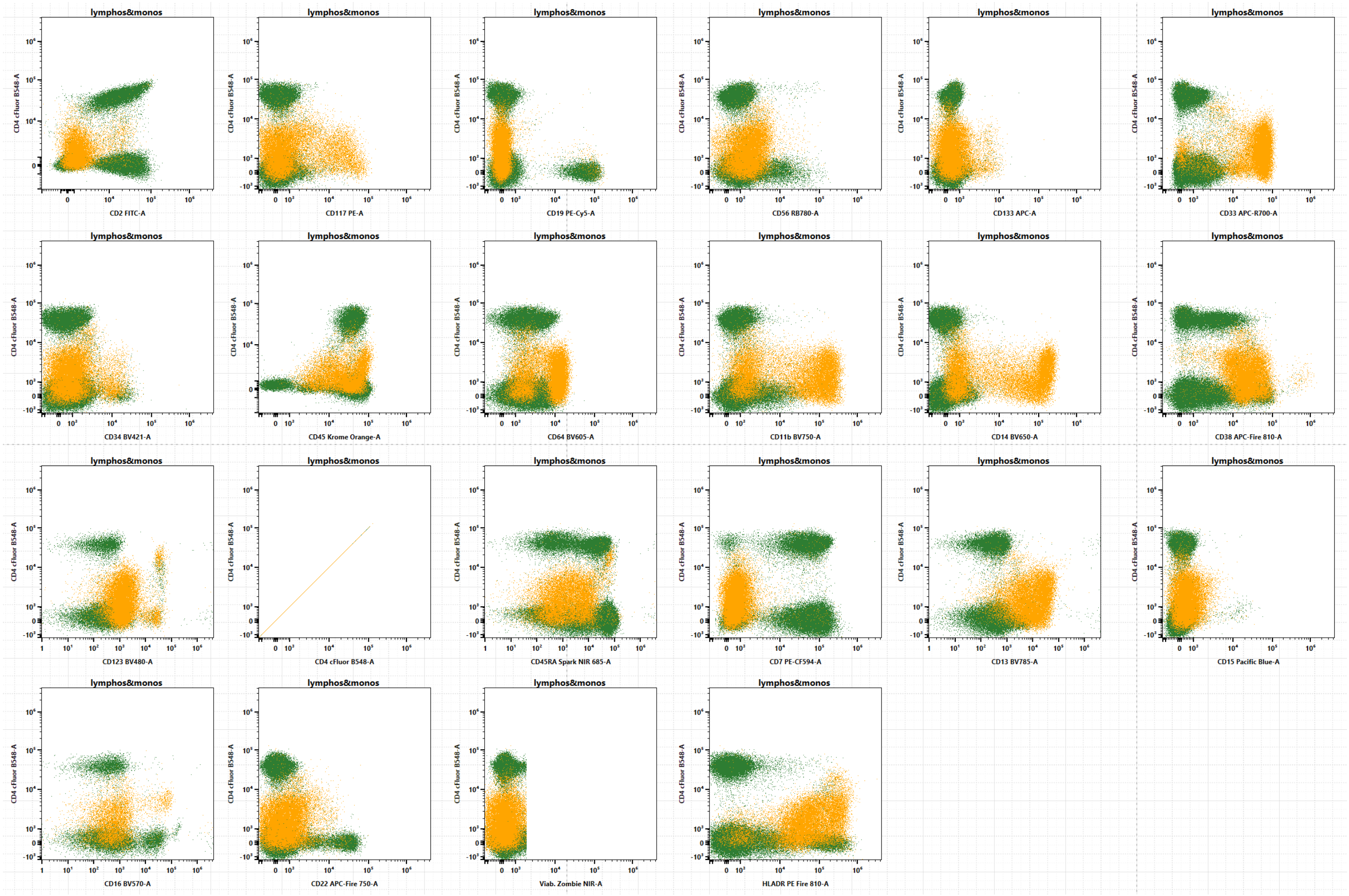
lymphos&monos
CD4 cFluor B548-A
CD2 FITC-A
CD117 PE-A
CD19 PE-Cy5-A
CD56 RB780-A
CD133 APC-A
CD33 APC-R700-A
CD34 BV421-A
CD45 Krome Orange-A
CD64 BV605-A
CD11b BV750-A
CD14 BV650-A
CD38 APC-Fire 810-A
CD123 BV480-A
CD4 cFluor B548-A
CD45RA Spark NIR 685-A
CD7 PE-CF594-A
CD13 BV785-A
CD15 Pacific Blue-A
CD16 BV570-A
CD22 APC-Fire 750-A
Viab. Zombie NIR-A
HLADR PE Fire 810-A

CD14

BM:

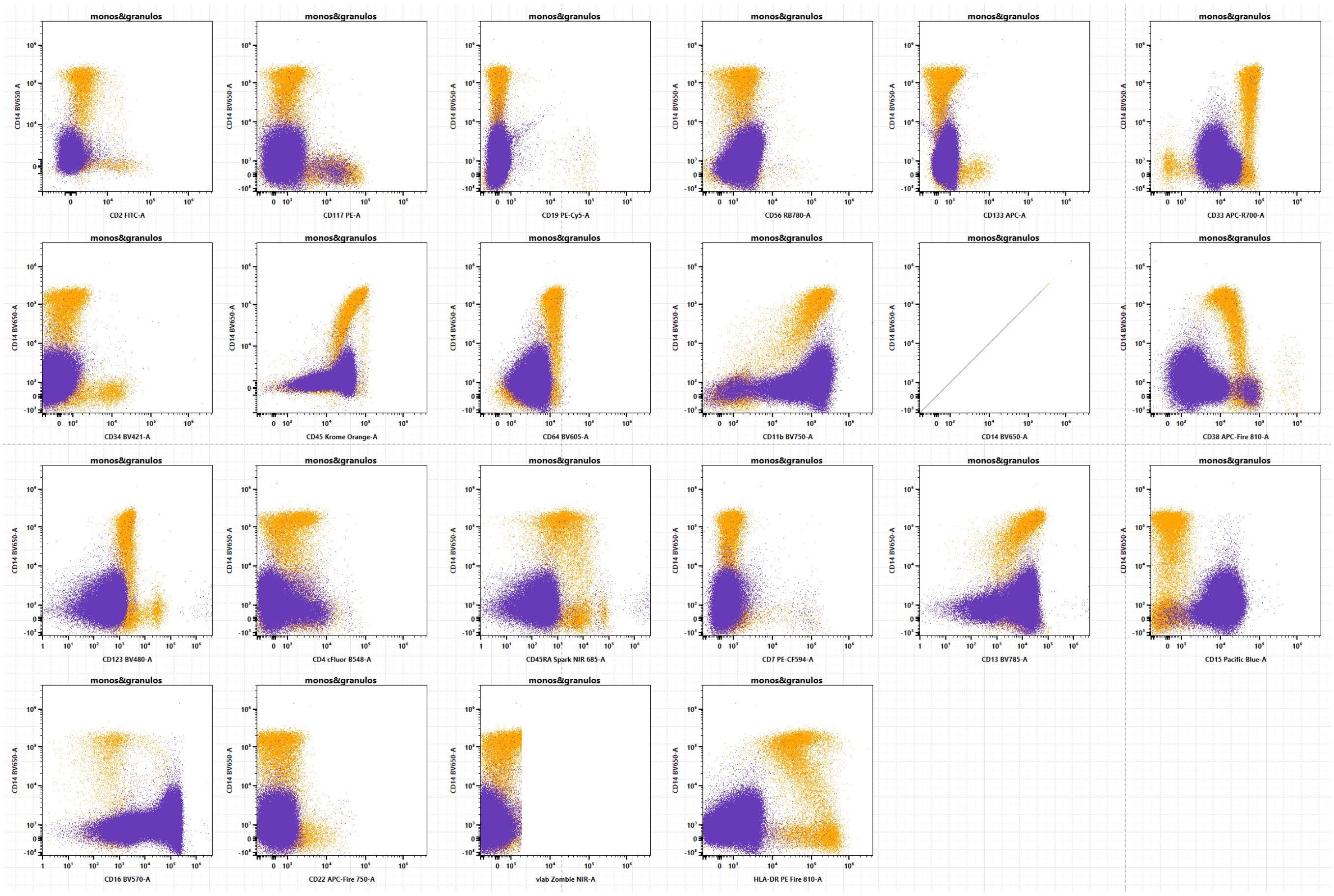
monos&granulos
CD14 BV650-A
CD2 FITC-A
CD117 PE-A
CD19 PE-Cy5-A
CD56 RB780-A
CD133 APC-A
CD33 APC-R700-A
CD34 BV421-A
CD45 Krome Orange-A
CD64 BV605-A
CD11b BV750-A
CD14 BV650-A
CD38 APC-Fire 810-A
CD123 BV480-A
CD4 cFluor B548-A
CD45RA Spark NIR 685-A
CD7 PE-CF594-A
CD13 BV785-A
CD15 Pacific Blue-A
CD16 BV570-A
CD22 APC-Fire 750-A
viab Zombie NIR-A
HLA-DR PE Fire 810-A

PB:

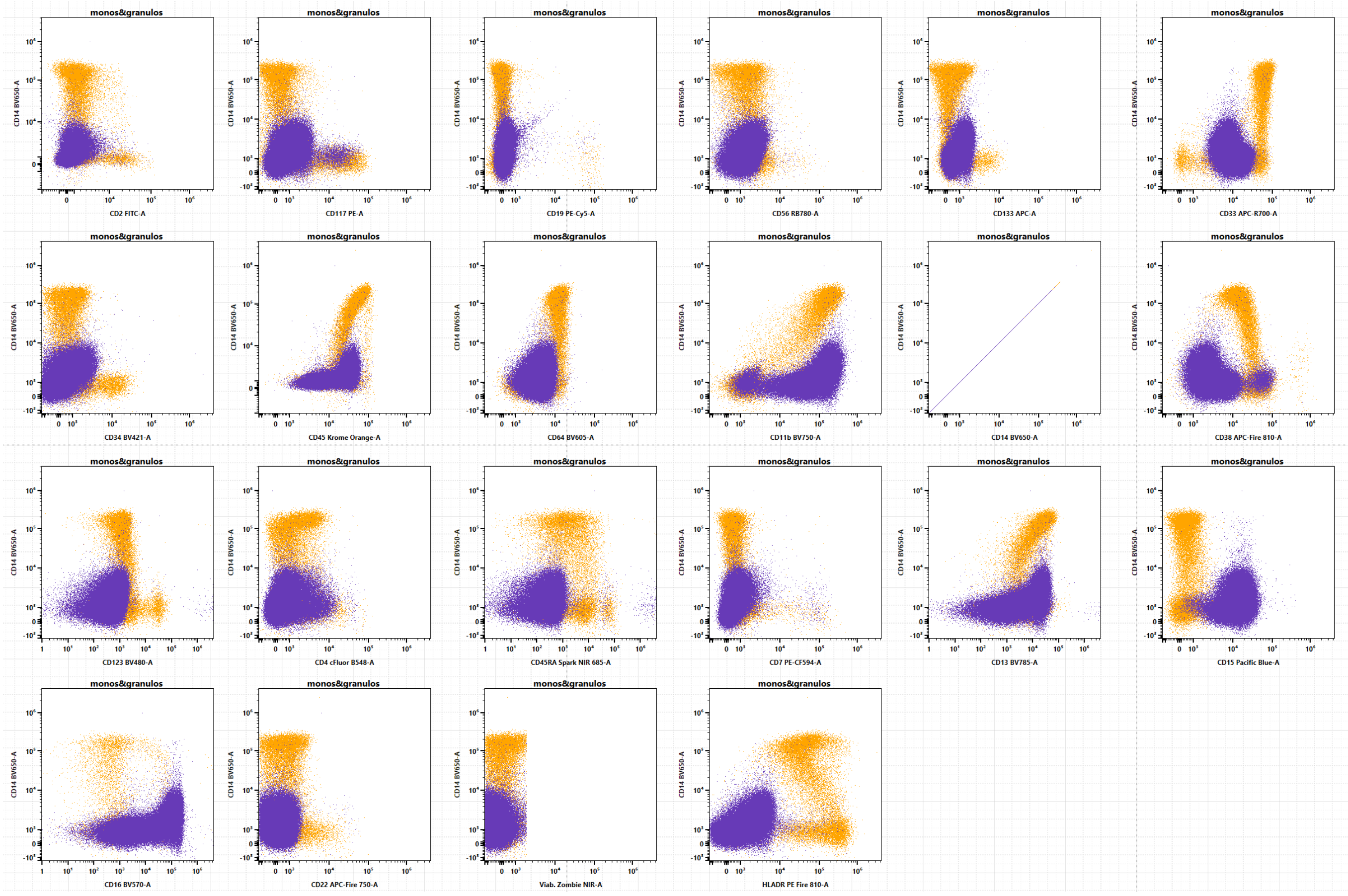
monos&granulos
CD14 BV650-A
CD2 FITC-A
CD117 PE-A
CD19 PE-Cy5-A
CD56 RB780-A
CD133 APC-A
CD33 APC-R700-A
CD34 BV421-A
CD45 Krome Orange-A
CD64 BV605-A
CD11b BV750-A
CD14 BV650-A
CD38 APC-Fire 810-A
CD123 BV480-A
CD4 cFluor B548-A
CD45RA Spark NIR 685-A
CD7 PE-CF594-A
CD13 BV785-A
CD15 Pacific Blue-A
CD16 BV570-A
CD22 APC-Fire 750-A
Viab. Zombie NIR-A
HLADR PE Fire 810-A

CD16

BM:

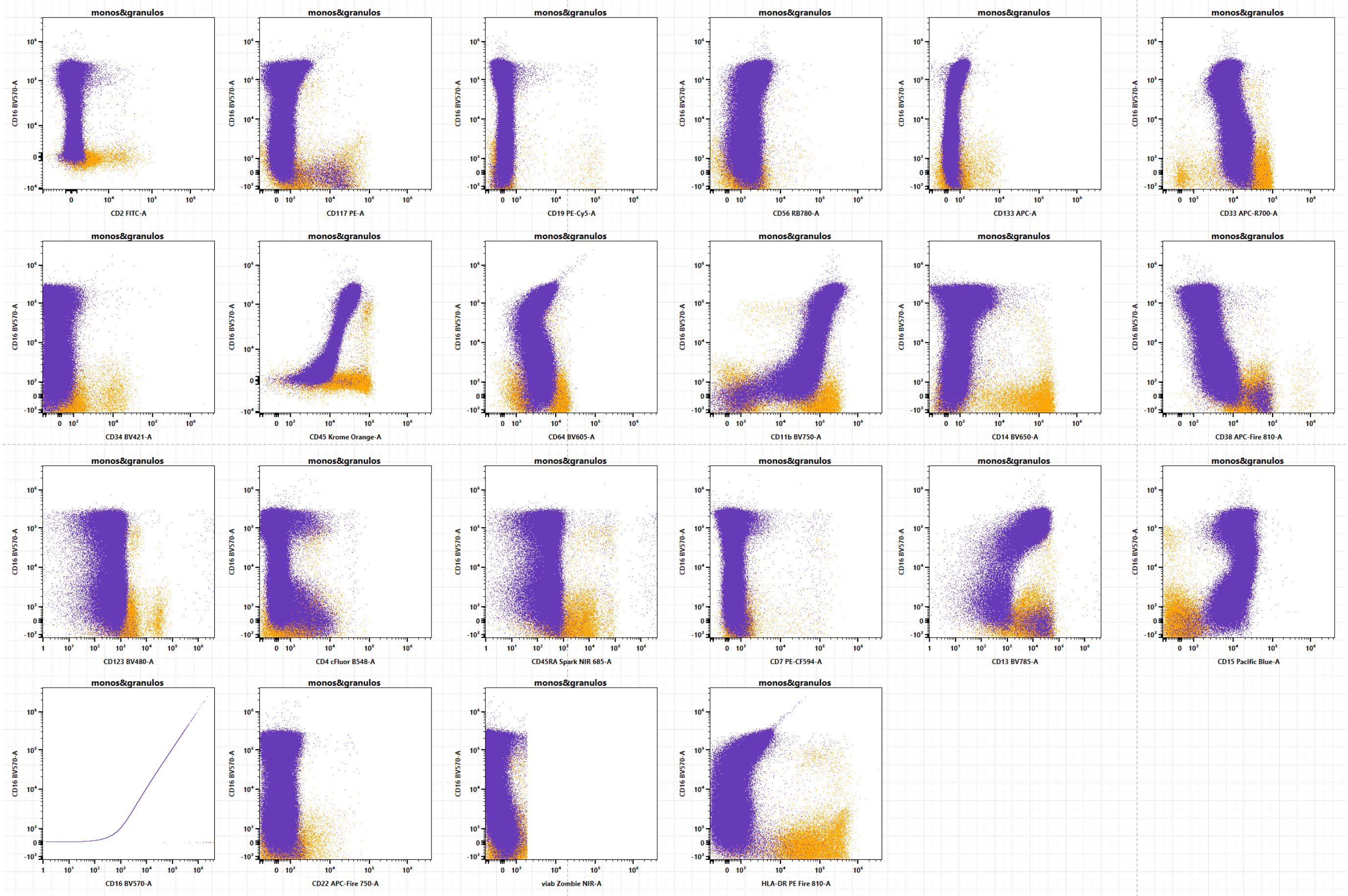
monos&granulos
CD16 BV570-A
CD2 FITC-A
CD117 PE-A
CD19 PE-Cy5-A
CD56 RB780-A
CD133 APC-A
CD33 APC-R700-A
CD34 BV421-A
CD45 Krome Orange-A
CD64 BV605-A
CD11b BV750-A
CD14 BV650-A
CD38 APC-Fire 810-A
CD123 BV480-A
CD4 cFluor B548-A
CD45RA Spark NIR 685-A
CD7 PE-CF594-A
CD13 BV785-A
CD15 Pacific Blue-A
CD16 BV570-A
CD22 APC-Fire 750-A
viab Zombie NIR-A
HLA-DR PE Fire 810-A

PB:

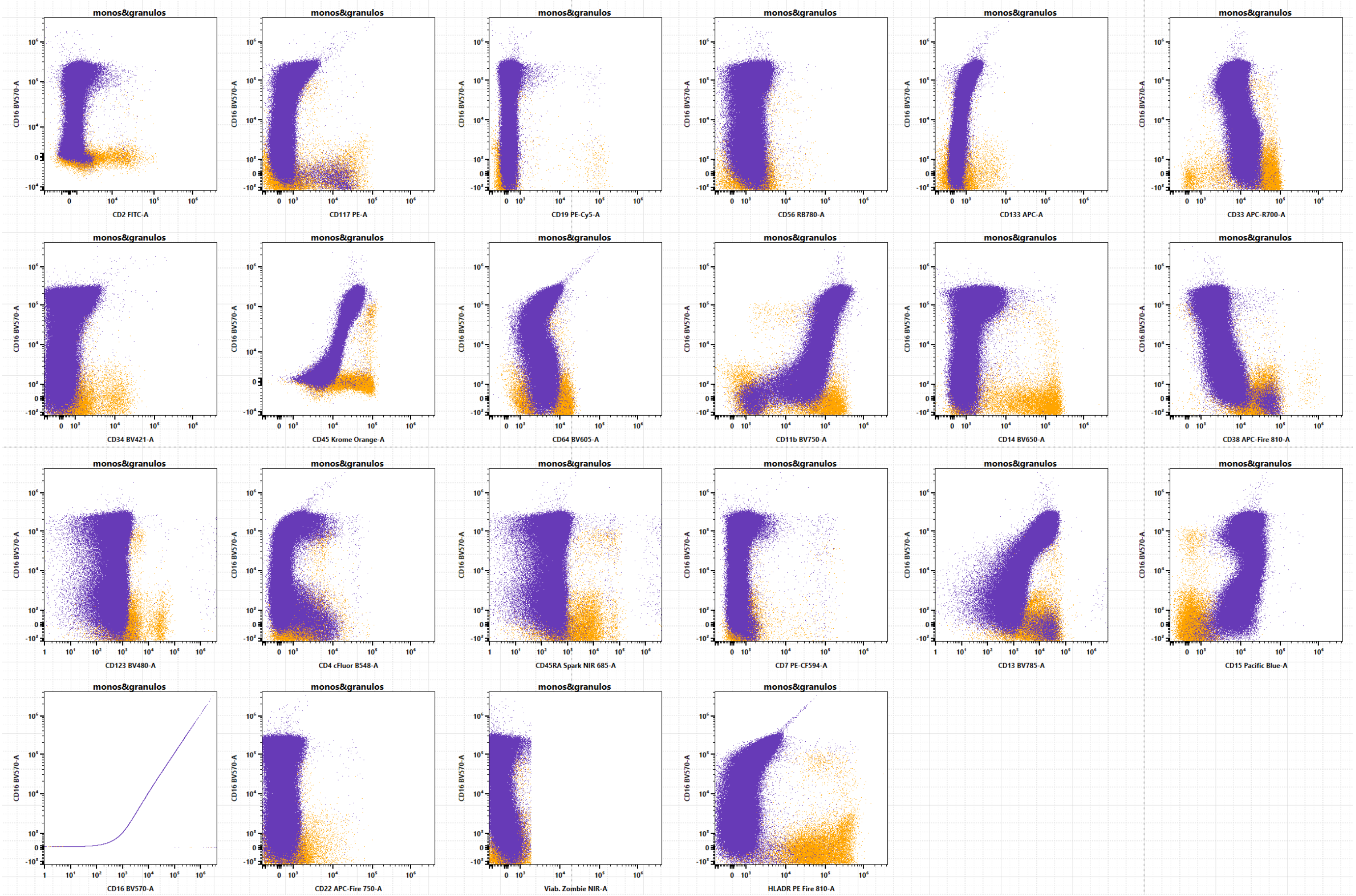
monos&granulos
CD16 BV570-A
CD2 FITC-A
CD117 PE-A
CD19 PE-Cy5-A
CD56 RB780-A
CD133 APC-A
CD33 APC-R700-A
CD34 BV421-A
CD45 Krome Orange-A
CD64 BV605-A
CD11b BV750-A
CD14 BV650-A
CD38 APC-Fire 810-A
CD123 BV480-A
CD4 cFluor B548-A
CD45RA Spark NIR 685-A
CD7 PE-CF594-A
CD13 BV785-A
CD15 Pacific Blue-A
CD16 BV570-A
CD22 APC-Fire 750-A
Viab. Zombie NIR-A
HLADR PE Fire 810-A

E

CD64

BM:

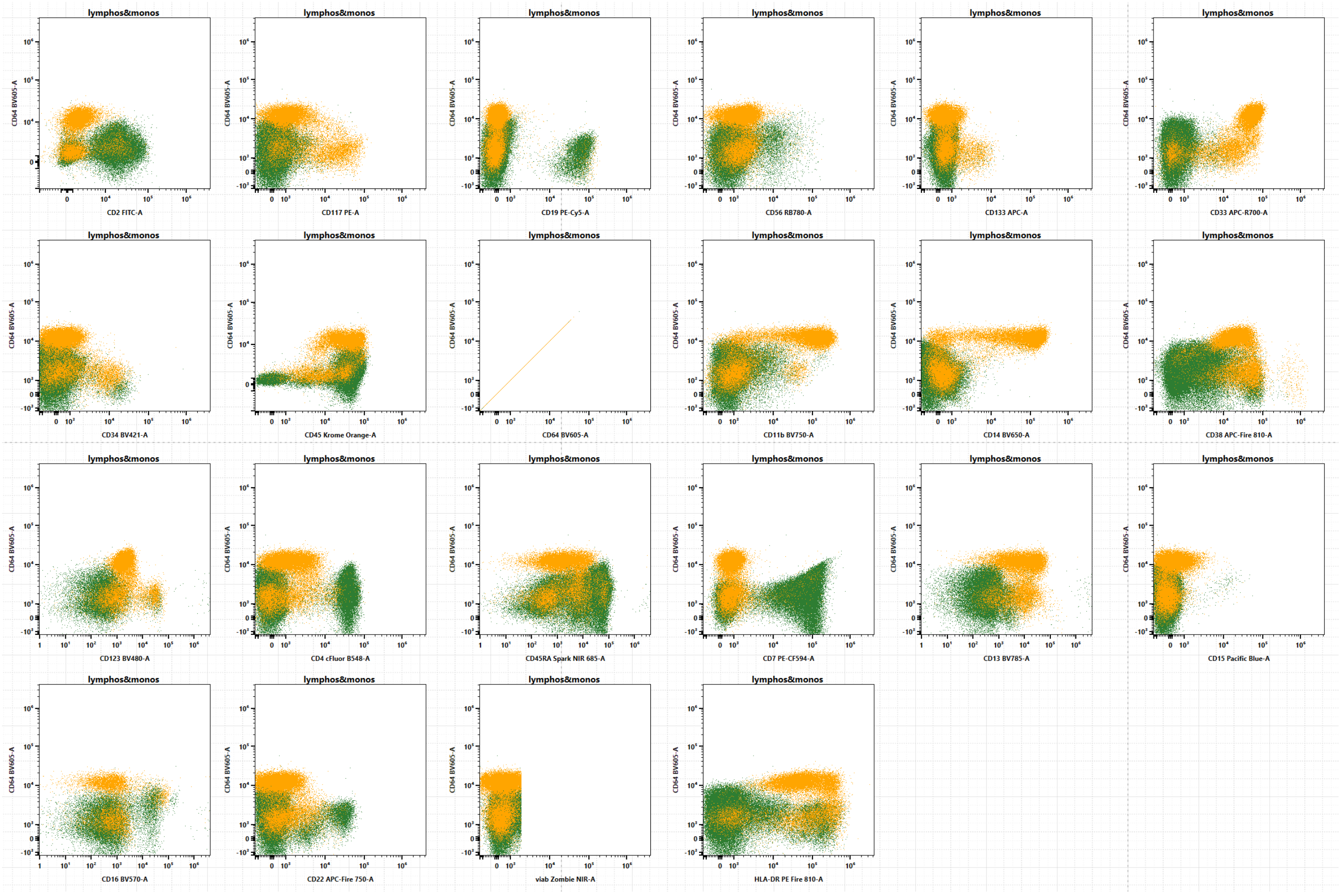
lymphos&monos
CD64 BV605-A
CD2 FITC-A
CD117 PE-A
CD19 PE-Cy5-A
CD56 RB780-A
CD133 APC-A
CD33 APC-R700-A
CD34 BV421-A
CD45 Krome Orange-A
CD64 BV605-A
CD11b BV750-A
CD14 BV650-A
CD38 APC-Fire 810-A
CD123 BV480-A
CD4 cFluor B548-A
CD45RA Spark NIR 685-A
CD7 PE-CF594-A
CD13 BV785-A
CD15 Pacific Blue-A
CD16 BV570-A
CD22 APC-Fire 750-A
viab Zombie NIR-A
HLA-DR PE Fire 810-A

PB:

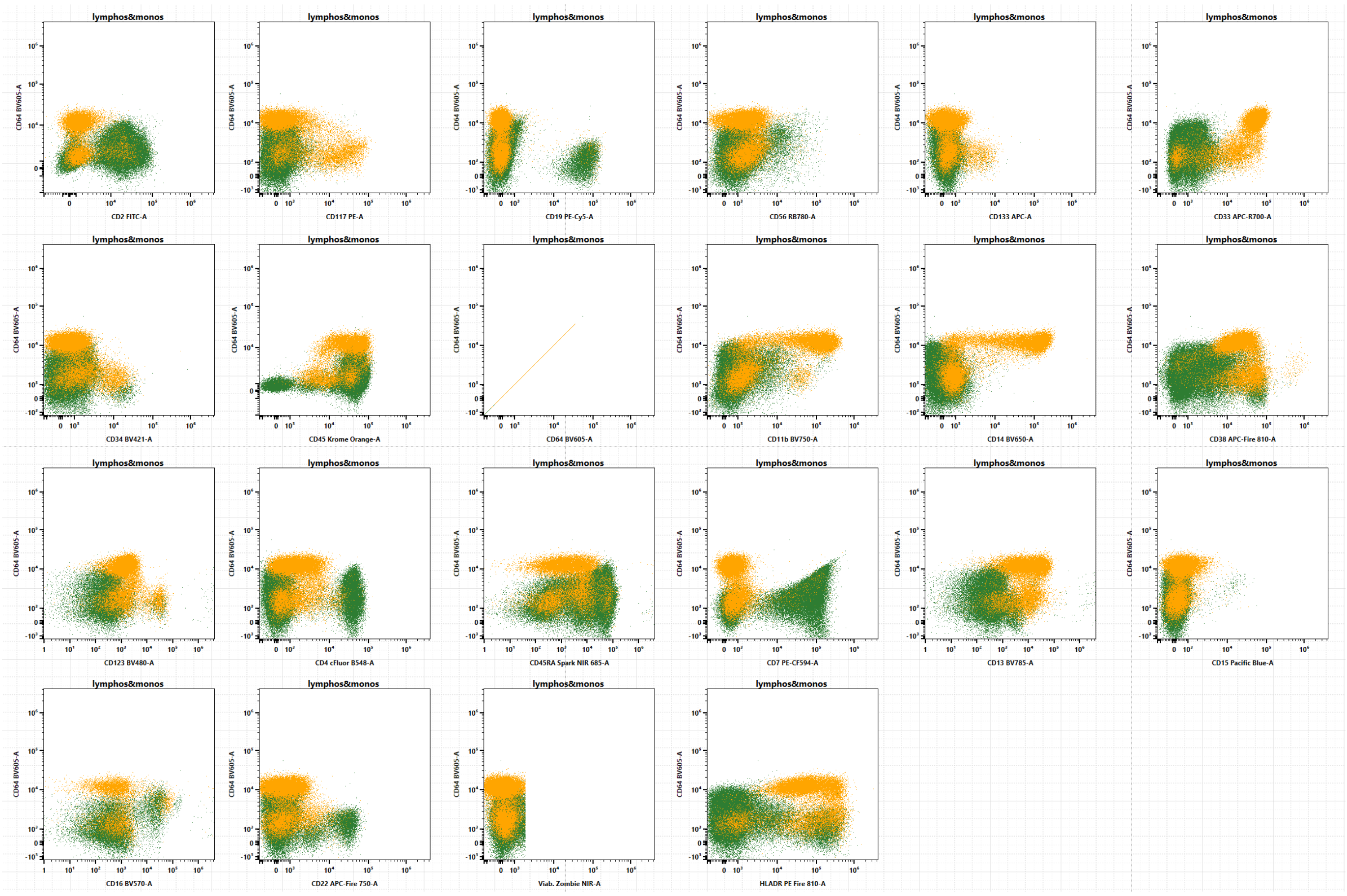
lymphos&monos
CD64 BV605-A
CD2 FITC-A
CD117 PE-A
CD19 PE-Cy5-A
CD56 RB780-A
CD133 APC-A
CD33 APC-R700-A
CD34 BV421-A
CD45 Krome Orange-A
CD64 BV605-A
CD11b BV750-A
CD14 BV650-A
CD38 APC-Fire 810-A
CD123 BV480-A
CD4 cFluor B548-A
CD45RA Spark NIR 685-A
CD7 PE-CF594-A
CD13 BV785-A
CD15 Pacific Blue-A
CD16 BV570-A
CD22 APC-Fire 750-A
Viab. Zombie NIR-A
HLADR PE Fire 810-A

F

CD34

BM:

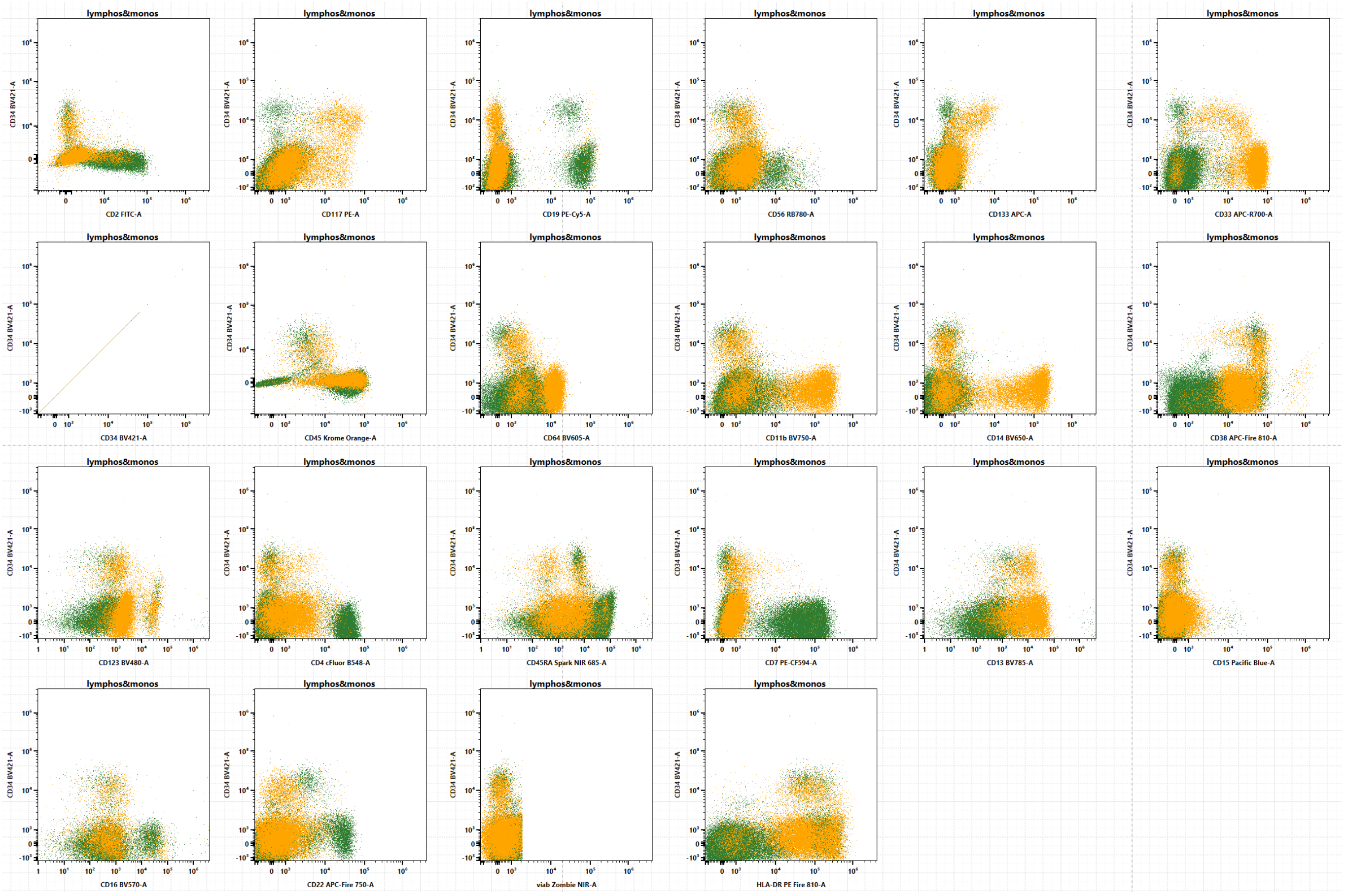

lymphos&monos
CD34 BV421-A
CD2 FITC-A
CD117 PE-A
CD19 PE-Cy5-A
CD56 RB780-A
CD133 APC-A
CD33 APC-R700-A
CD34 BV421-A
CD45 Krome Orange-A
CD64 BV605-A
CD11b BV750-A
CD14 BV650-A
CD38 APC-Fire 810-A
CD123 BV480-A
CD4 cFluor B548-A
CD45RA Spark NIR 685-A
CD7 PE-CF594-A
CD13 BV785-A
CD15 Pacific Blue-A
CD16 BV570-A
CD22 APC-Fire 750-A
viab Zombie NIR-A
HLA-DR PE Fire 810-A

PB:

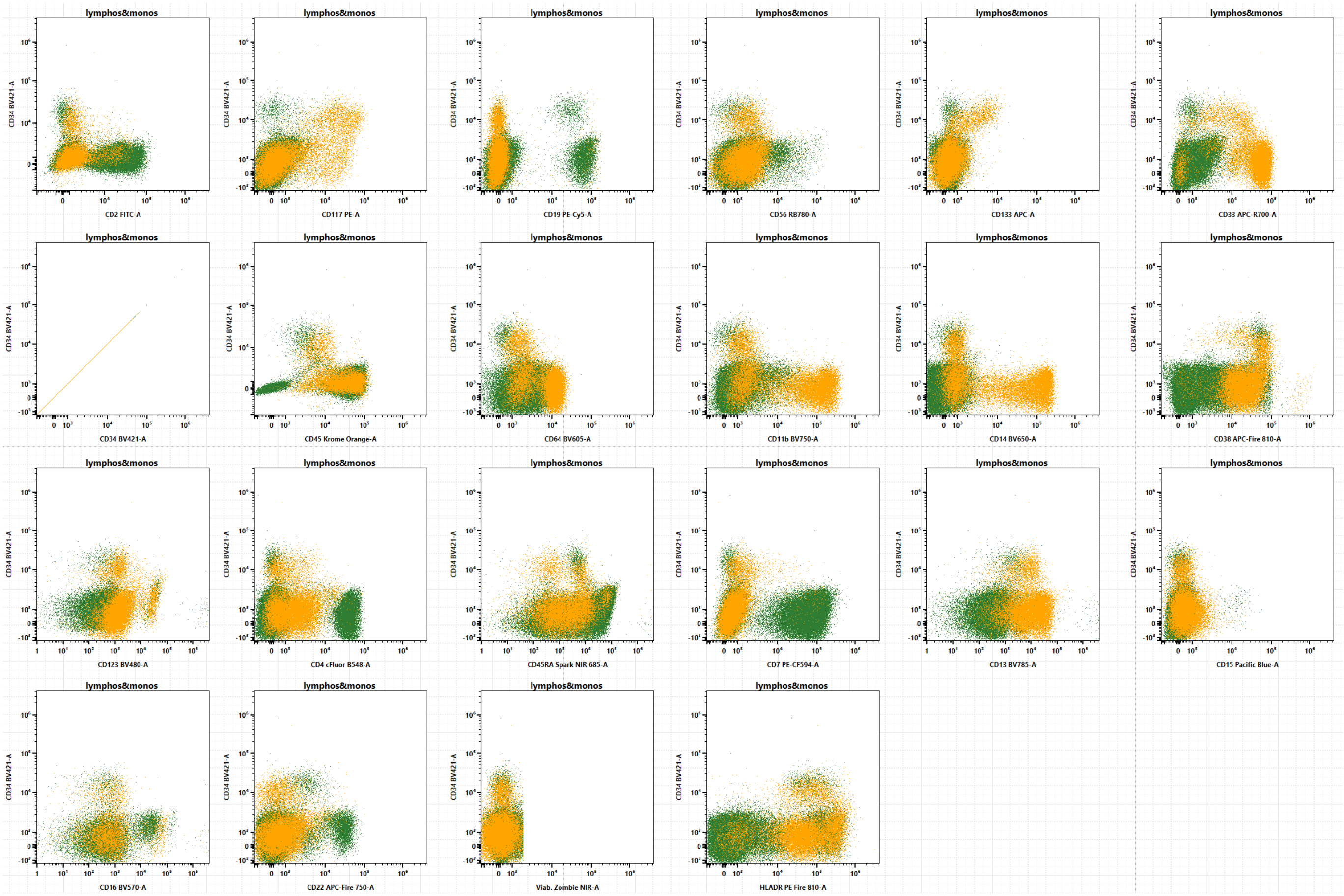

lymphos&monos
CD34 BV421-A
CD2 FITC-A
CD117 PE-A
CD19 PE-Cy5-A
CD56 RB780-A
CD133 APC-A
CD33 APC-R700-A
CD34 BV421-A
CD45 Krome Orange-A
CD64 BV605-A
CD11b BV750-A
CD14 BV650-A
CD38 APC-Fire 810-A
CD123 BV480-A
CD4 cFluor B548-A
CD45RA Spark NIR 685-A
CD7 PE-CF594-A
CD13 BV785-A
CD15 Pacific Blue-A
CD16 BV570-A
CD22 APC-Fire 750-A
Viab. Zombie NIR-A
HLADR PE Fire 810-A

CD117
BM:

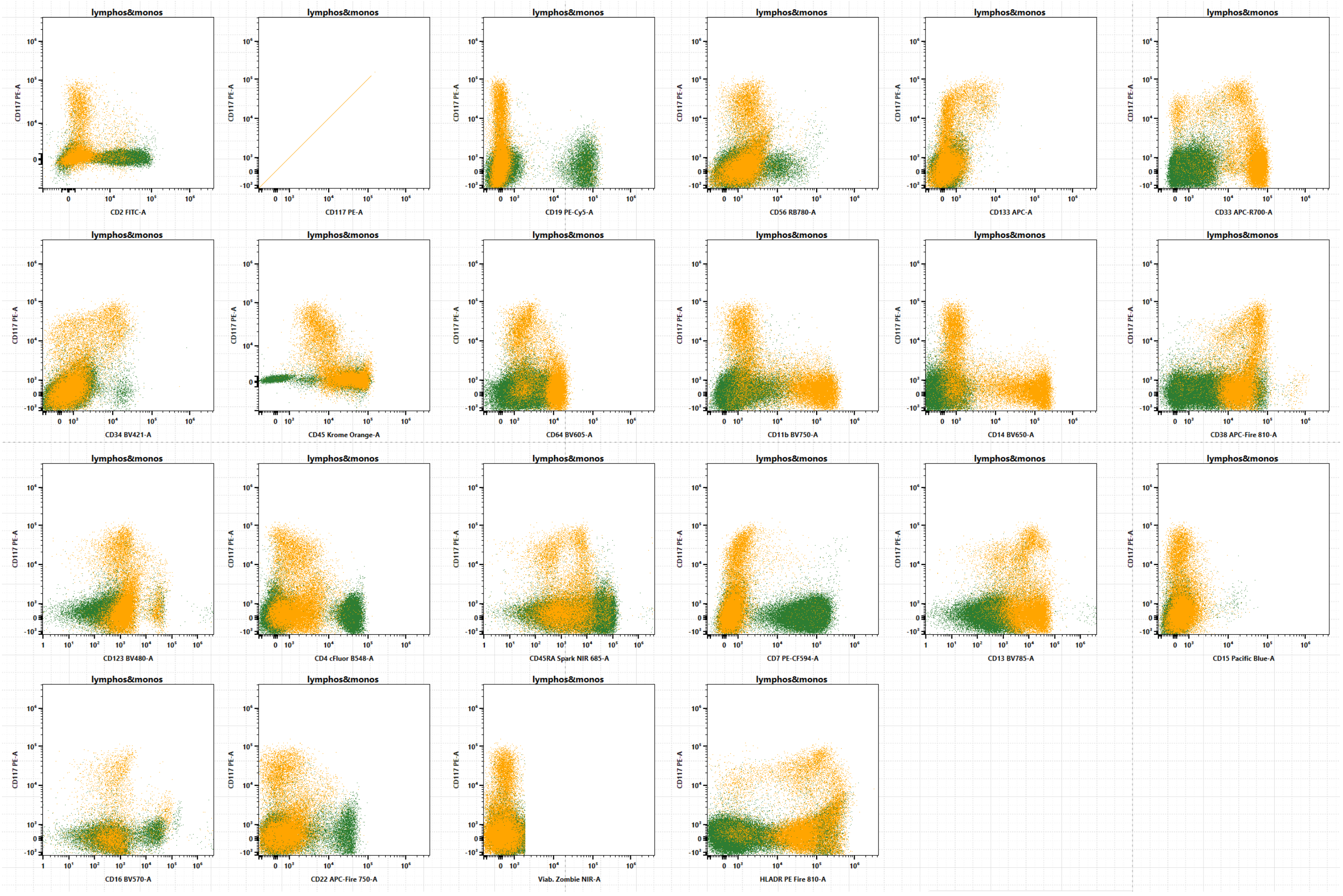
lymphos&monos
CD117 PE-A
CD2 FITC-A
CD117 PE-A
CD19 PE-Cy5-A
CD56 RB780-A
CD133 APC-A
CD33 APC-R700-A
CD34 BV421-A
CD45 Krome Orange-A
CD64 BV605-A
CD11b BV750-A
CD14 BV650-A
CD38 APC-Fire 810-A
CD123 BV480-A
CD4 cFluor B548-A
CD45RA Spark NIR 685-A
CD7 PE-CF594-A
CD13 BV785-A
CD15 Pacific Blue-A
CD16 BV570-A
CD22 APC-Fire 750-A
Viab. Zombie NIR-A
HLADR PE Fire 810-A

PB:

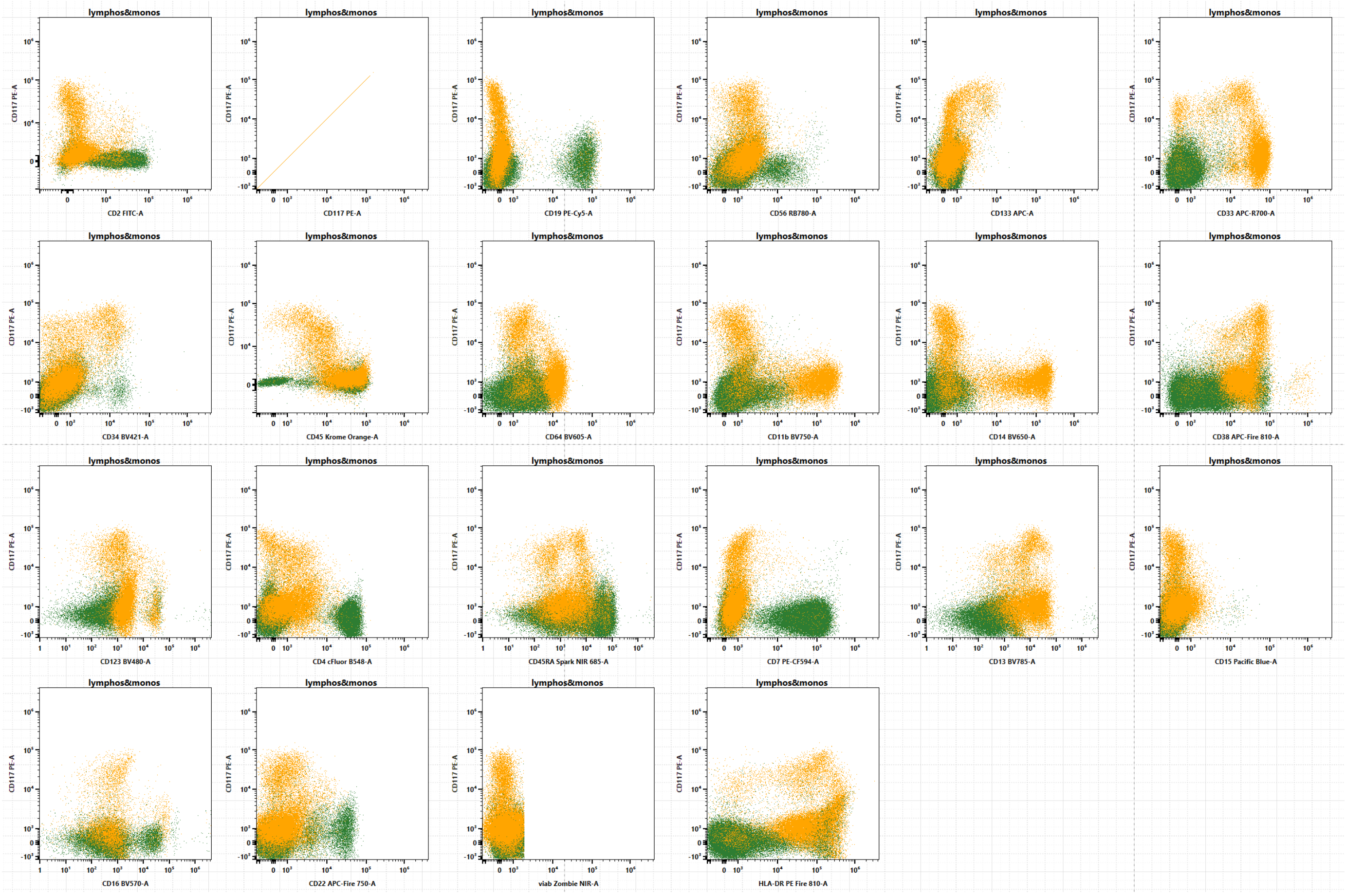
lymphos&monos
CD117 PE-A
CD2 FITC-A
CD117 PE-A
CD19 PE-Cy5-A
CD56 RB780-A
CD133 APC-A
CD33 APC-R700-A
CD34 BV421-A
CD45 Krome Orange-A
CD64 BV605-A
CD11b BV750-A
CD14 BV650-A
CD38 APC-Fire 810-A
CD123 BV480-A
CD4 cFluor B548-A
CD45RA Spark NIR 685-A
CD7 PE-CF594-A
CD13 BV785-A
CD15 Pacific Blue-A
CD16 BV570-A
CD22 APC-Fire 750-A
viab Zombie NIR-A
HLA-DR PE Fire 810-A

CD133

BM:

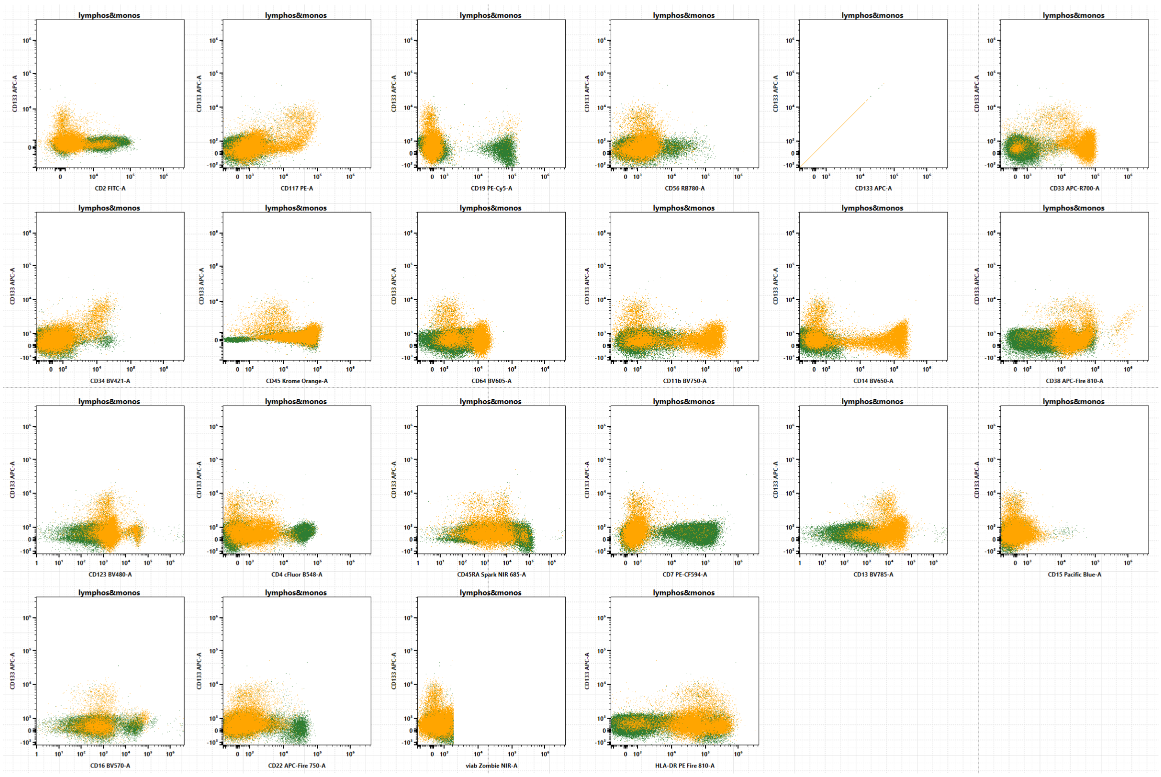

PB:

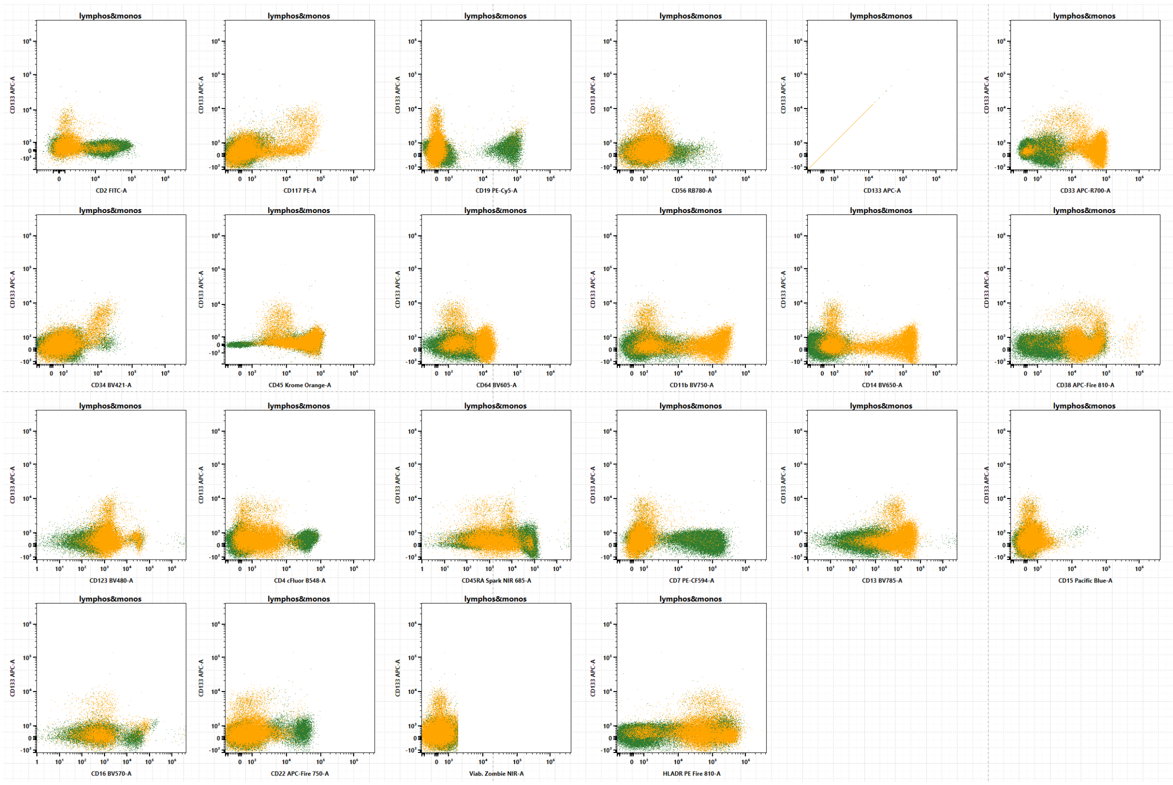

Figure S3: Comparison of nBM cell-line unmixing with PB surrogate unmixing

A nBM sample was stained with the 22-color panel and unmixed through the nBM cell-line approach (from Fokken et al., 2024, https://pubmed.ncbi.nlm.nih.gov/37984809/) and the PB surrogate approach (described in results section 3.2) respectively. Throughout this figure nBM cell-line unmixed data (BM) are always shown on the top and PB surrogate unmixed data (PB) on the bottom of the subfigures. In subfigure **(A)** only unstained data are shown. In subfigures **(B) – (F)** only data, which are already unmixed in SpectroFlo, through the nBM cell-line approach and the PB surrogate approach are shown. **(A)** Comparison of autofluorescence spectra of major WBC populations. **(B)** Compensation matrices. **(C)** Gating of Major WBC populations. **(D)** NxN plots for characteristic markers representative of major WBC populations (CD4 lymphocytes, CD14 monocytes, CD16 granulocytes). **(E)** NxN plots for CD64. **(F)** NxN plots for primitive markers CD34, CD117, CD133.

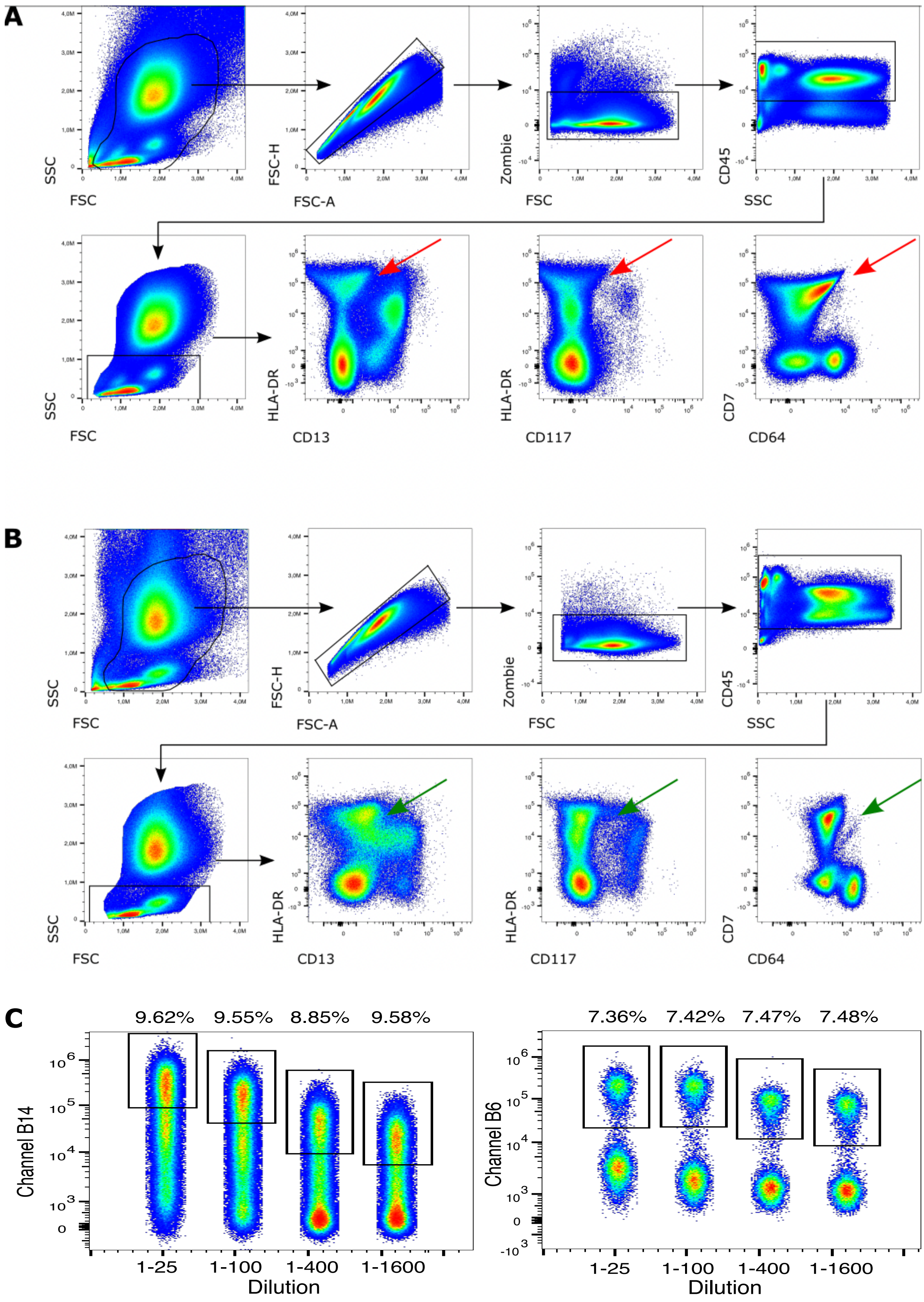

Figure S4: Optimized antibody concentrations reduce data spread and increase data resolution.

(A) Bone marrow samples were stained with the 22-color panel, containing antibody concentrations that provided optimal signal-to-noise ratios in titration experiments. When gating for SSC^{low} (lymphocytes, monocytes, blasts), data spread became especially strong for HLA-DR vs CD13, HLA-DR vs CD117, and CD7 vs CD64. (**B**) The staining of bone marrow cells was repeated with adjusted antibody concentrations that provided suboptimal signal-to-noise ratios in titration experiments. However, due to reduced signal intensities, the data spread could be reduced, resulting in overall better separation between the populations. **(C)** Dilution of HLA-DR-PE-Fire-810 (left plot) and CD7-PE-CF594 (right plot) on bone marrow cells, plotted against each fluorochromes peak channel (B14 for PE-Fire-810 and B6 for PE-CF594). The frequency of gated events per dilution (25% of all concatenated cells) is indicated at the top of each plot.

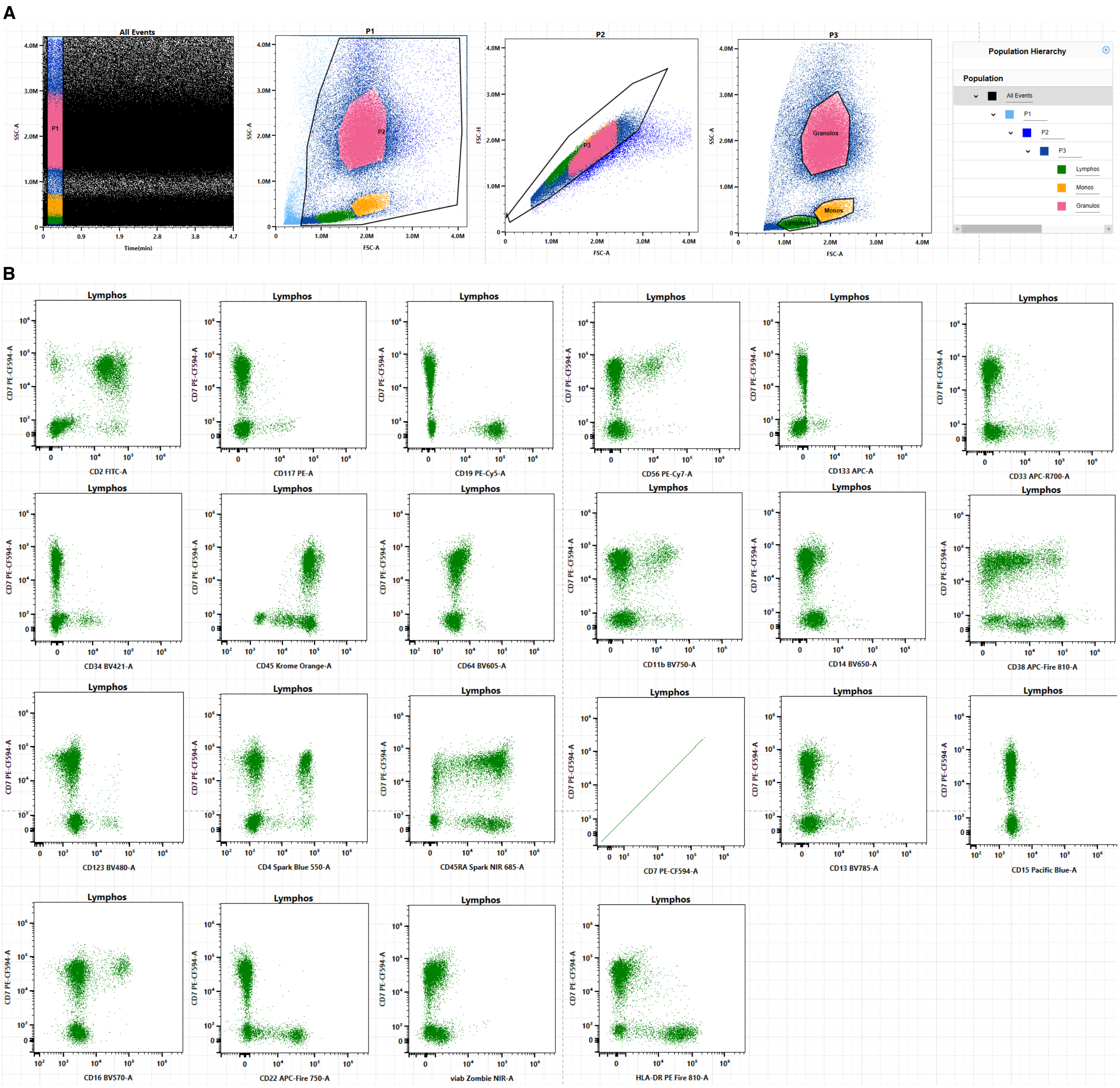
A
All Events
P1
P2
P3
SSC-A
FSC-A
FSC-H
Time(min)
Granulos
Monos
Population Hierarchy
Population
All Events
P1
P2
P3
Lymphos
Monos
Granulos
B
Lymphos
CD7 PE-CF594-A
CD2 FITC-A
CD117 PE-A
CD19 PE-Cy5-A
CD56 PE-Cy7-A
CD133 APC-A
CD33 APC-R700-A
CD34 BV421-A
CD45 Krome Orange-A
CD64 BV605-A
CD11b BV750-A
CD14 BV650-A
CD38 APC-Fire 810-A
CD123 BV480-A
CD4 Spark Blue 550-A
CD45RA Spark NIR 685-A
CD7 PE-CF594-A
CD13 BV785-A
CD15 Pacific Blue-A
CD16 BV570-A
CD22 APC-Fire 750-A
viab Zombie NIR-A
HLA-DR PE Fire 810-A

C

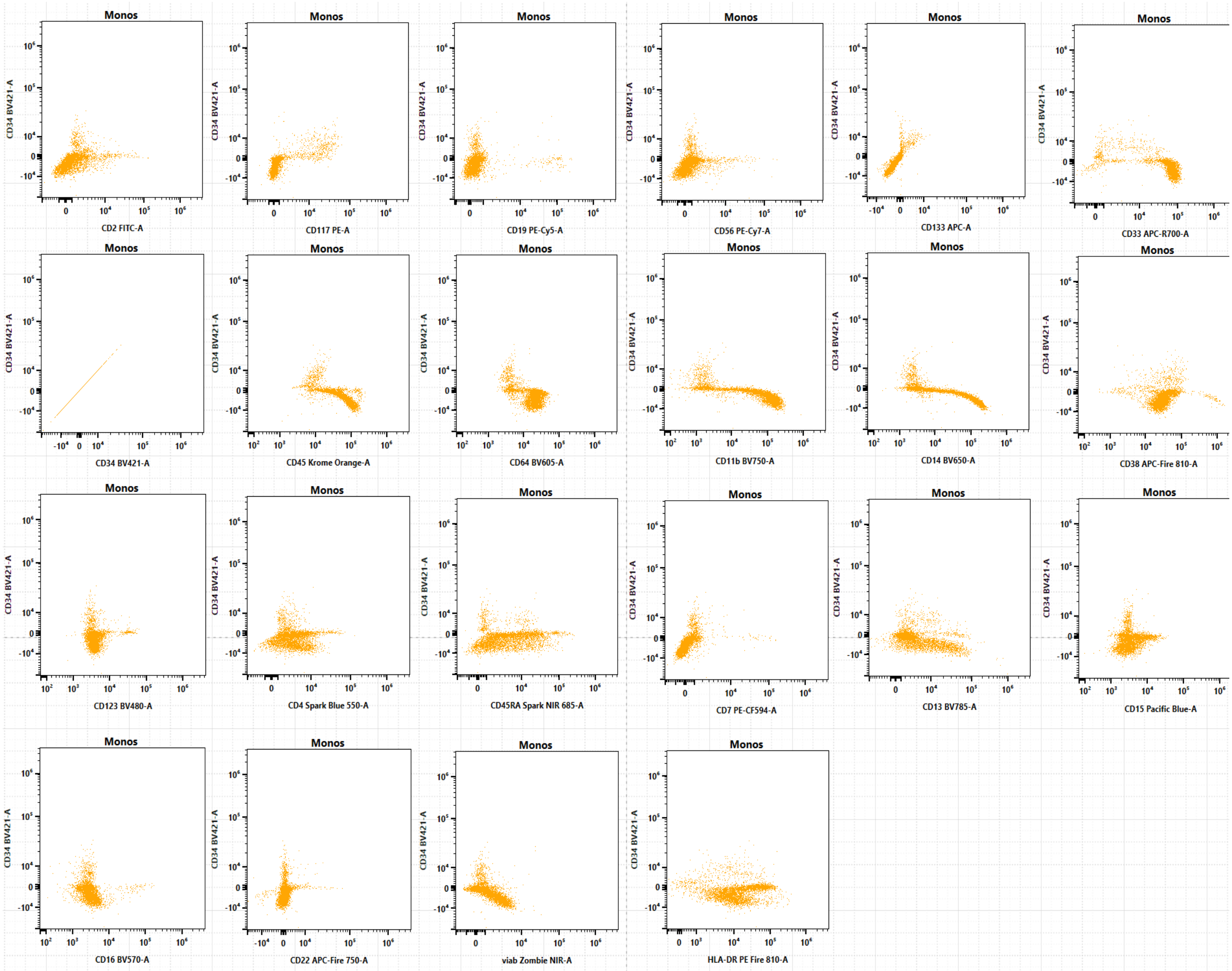

D

INTO ↓ FROM →	BV421	Pacific Blue	BV480	Krome Orange	BV570	BV605	BV650	BV750	BV785	FITC	Spark Blue 550	PE	PE-CF594	PE-Cy5	PE-Cy7	PE Fire 810	APC	Spark NIR 685	APC-R700	Zombie NIR	APC-Fire 750	APC-Fire 810
BV421	100	0	0	0	0	0	0	0	0	0	0	0	0	0	0	0	0	0	0	0	0	0
Pacific Blue	0	100	0	0	0	0	-1.78	0	0	0	0	0	0	0	0	0	0	0	0	0	0	0
BV480	0	0	100	0	0	0	0	0	0	0	0	0	0	0	0	0	0	0	0	0	0	0
Krome Orange	0	0	0	100	0	0	0	0	0	0	0	0	0	0	0	0	0	0	0	0	0	0
BV570	0	0	0	0	100	0	0	0	0	0	0	0	0	0	0	0	0	0	0	0	0	0
BV605	0	0	0	0	0	100	0	0	0	0	0	0	0	0	0	0	0	0	0	0	0	0
BV650	0	0	0	0	0	0	100	0	0	0	0	0	0	0	0	0	0	0	0	0	0	0
BV750	0	0	0	0	0	0	0	100	0	0	0	0	0	0	0	0	0	0	0	0	0	0
BV785	0	0	0	0	0	0	0	0	100	0	0	0	0	0	0	0	0	0	0	0	0	0
FITC	0	0	0	0	0	0	0	0	0	100	0	0	0	0	0	0	0	0	0	0	0	0
Spark Blue 550	0	0	0	0	0	0	0	0	0	0	100	0	0	0	0	0	0	0	0	0	0	0
PE	0	0	0	0	0	0	-1.5	0	0	0	0	100	0	0	0	0	0	0	0	0	0	0
PE-CF594	0	0	0	0	0	0	-1.05	0	0	0	0	0	100	0	0	0	0	0	0	0	0	0
PE-Cy5	0	0	0	0	0	0	0	0	0	0	0	0	0	100	0	0	0	0	0	0	0	0
PE-Cy7	0	0	0	0	0	0	0	0	0	0	0	0	0	0	100	0	0	0	0	0	0	0
PE Fire 810	0	0	0	0	0	0	0	0	0	0	0	0	0	0	0	100	0	0	0	0	0	0
APC	0	0	0	0	0	0	-3	0	0	0	0	0	0	0	0	0	100	0	0	0	0	0
Spark NIR 685	0	0	0	0	0	0	0	0	0	0	0	0	0	0	0	0	0	100	0	0	0	0
APC-R700	0	0	0	0	0	0	0	0	0	0	0	0	0	0	0	0	0	0	100	0	0	0
Zombie NIR	0	0	0	0	0	0	0	0	0	0	0	0	0	0	0	0	0	0	0	100	0	0
APC-Fire 750	0	0	0	0	0	0	0	0	0	0	0	0	0	0	0	0	0	0	0	0	100	0
APC-Fire 810	0	0	0	0	0	0	0	0	0	0	0	0	0	0	0	0	0	0	0	0	0	100

E

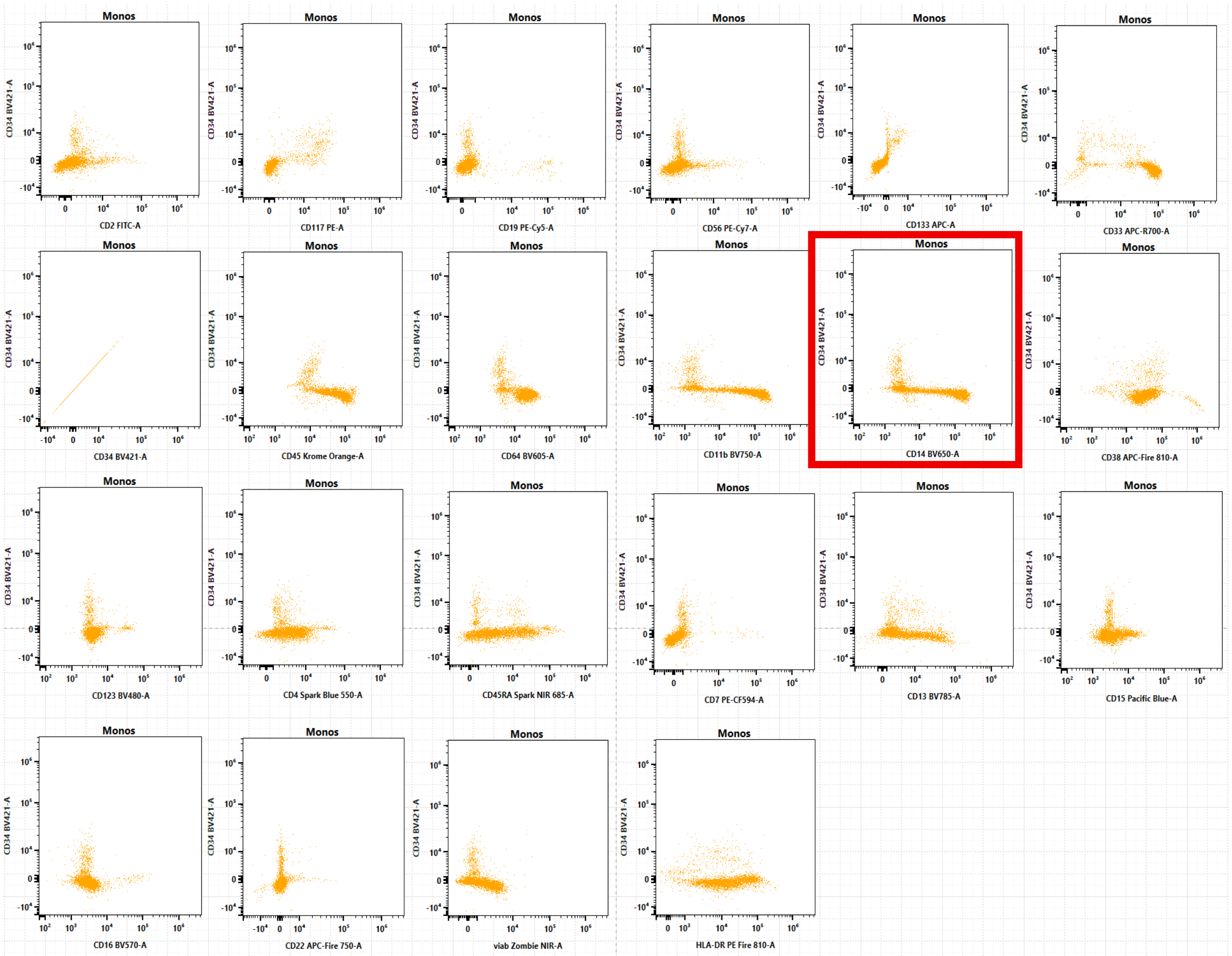

F

INTO ↓ FROM →	BV421	Pacific Blue	BV480	Krome Orange	BV570	BV605	BV650	BV750	BV785	FITC	Spark Blue 550	PE	PE-CF594	PE-Cy5	PE-Cy7	PE Fire 810	APC	Spark NIR 685	APC-R700	Zombie NIR	APC-Fire 750	APC-Fire 810
BV421	100	0	0	0	0	0	-3	0	0	0	0	0	0	0	0	0	0	0	0	0	0	0
Pacific Blue	0	100	0	0	0	0	-1.78	0	0	0	0	0	0	0	0	0	0	0	0	0	0	0
BV480	0	0	100	0	0	0	0	0	0	0	0	0	0	0	0	0	0	0	0	0	0	0
Krome Orange	0	0	0	100	0	0	0	0	0	0	0	0	0	0	0	0	0	0	0	0	0	0
BV570	0	0	0	0	100	0	0	0	0	0	0	0	0	0	0	0	0	0	0	0	0	0
BV605	0	0	0	0	0	100	0	0	0	0	0	0	0	0	0	0	0	0	0	0	0	0
BV650	0	0	0	0	0	0	100	0	0	0	0	0	0	0	0	0	0	0	0	0	0	0
BV750	0	0	0	0	0	0	0	100	0	0	0	0	0	0	0	0	0	0	0	0	0	0
BV785	0	0	0	0	0	0	0	0	100	0	0	0	0	0	0	0	0	0	0	0	0	0
FITC	0	0	0	0	0	0	0	0	0	100	0	0	0	0	0	0	0	0	0	0	0	0
Spark Blue 550	0	0	0	0	0	0	0	0	0	0	100	0	0	0	0	0	0	0	0	0	0	0
PE	0	0	0	0	0	0	-1.5	0	0	0	0	100	0	0	0	0	0	0	0	0	0	0
PE-CF594	0	0	0	0	0	0	-1.05	0	0	0	0	0	100	0	0	0	0	0	0	0	0	0
PE-Cy5	0	0	0	0	0	0	0	0	0	0	0	0	0	100	0	0	0	0	0	0	0	0
PE-Cy7	0	0	0	0	0	0	0	0	0	0	0	0	0	0	100	0	0	0	0	0	0	0
PE Fire 810	0	0	0	0	0	0	0	0	0	0	0	0	0	0	0	100	0	0	0	0	0	0
APC	0	0	0	0	0	0	-3	0	0	0	0	0	0	0	0	0	100	0	0	0	0	0
Spark NIR 685	0	0	0	0	0	0	0	0	0	0	0	0	0	0	0	0	0	100	0	0	0	0
APC-R700	0	0	0	0	0	0	0	0	0	0	0	0	0	0	0	0	0	0	100	0	0	0
Zombie NIR	0	0	0	0	0	0	0	0	0	0	0	0	0	0	0	0	0	0	0	100	0	0
APC-Fire 750	0	0	0	0	0	0	0	0	0	0	0	0	0	0	0	0	0	0	0	0	100	0
APC-Fire 810	0	0	0	0	0	0	0	0	0	0	0	0	0	0	0	0	0	0	0	0	0	100

Figure S5 – Accurate unmixing and manual compensation in SpectroFlo

(A) Gating of WBC, granulocytes, lmyphocytes, and monocytes in SpectroFlo. **(B)** Example of accurate unmixing of CD7 against all other markers of the 22-color panel (no compensation required). **(C)** Example of inaccurate unmixing between CD34-BV421 and multiple other fluorochromes resulting in negative MFIs in the BV421 channel. **(D)** Compensation matrix without correction for spillover of BV421. **(E)** Example of manually corrected spillover between CD34-BV421 and CD14-BV650 in 2D plot. **(F)** Compensation matrix with corrected spillover (3% correction).

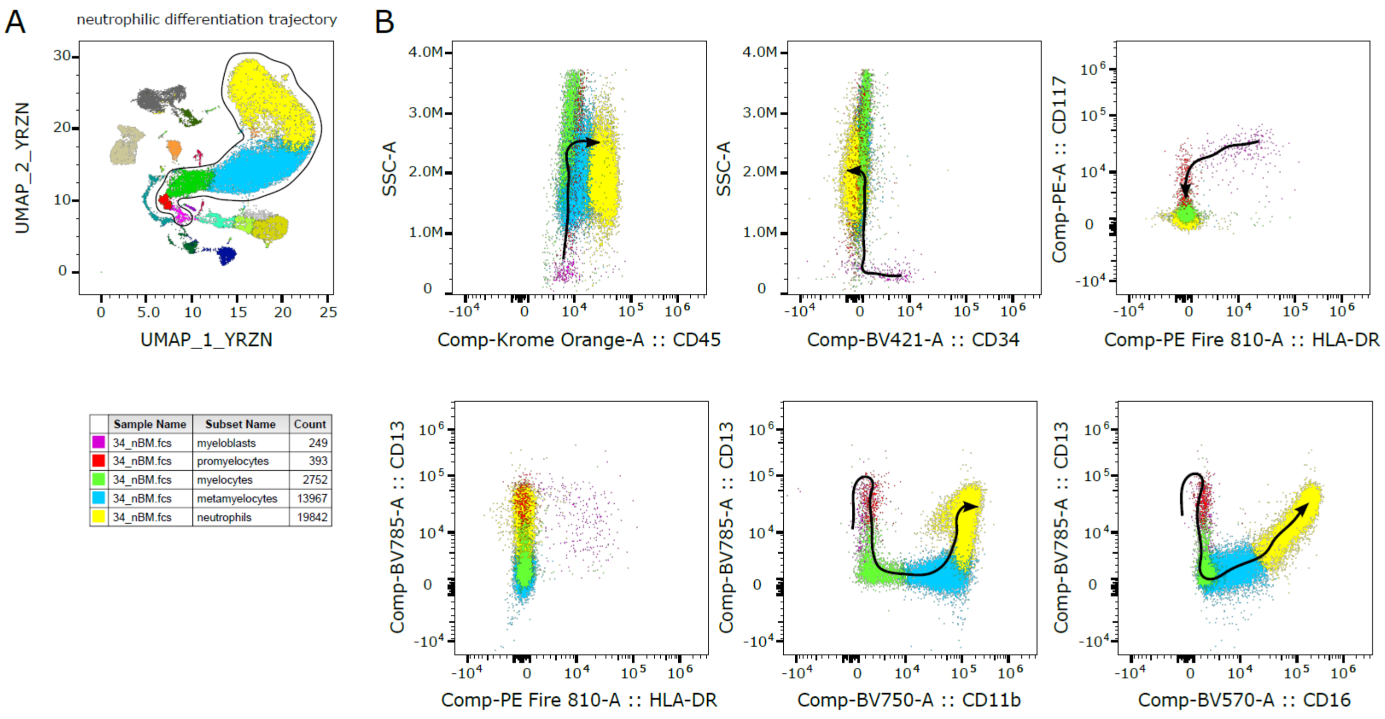

	Sample Name	Subset Name	Count
	34_nBM.fcs	myeloblasts	249
	34_nBM.fcs	promyelocytes	393
	34_nBM.fcs	myelocytes	2752
	34_nBM.fcs	metamyelocytes	13967
	34_nBM.fcs	neutrophils	19842

Figure S6: Myeloid differentiation trajectory within the high-dimensional data space and by manual gating.
(**A**) Identification of the neutrophilic differentiation trajectory within a UMAP of nBM and color-coded FlowSOM clusters (as in Figure 1). (**B**) 2D plots of typical marker combinations for the identification of myeloblasts (pink), promyelocytes (red), myelocytes (green), metamyelocytes (teal) and neutrophils (yellow) within the gated clusters of (A). Color codes are identical between (A) and (B). Note that FlowSOM did not originally include the promyelocyte cluster in (A). It was therefore separated from the myelocyte population based on its characteristic expression of CD13^{bright} and CD117^{+} within the 2D plots. Data representation in (B) was inspired by https://www.learnhaem.com.

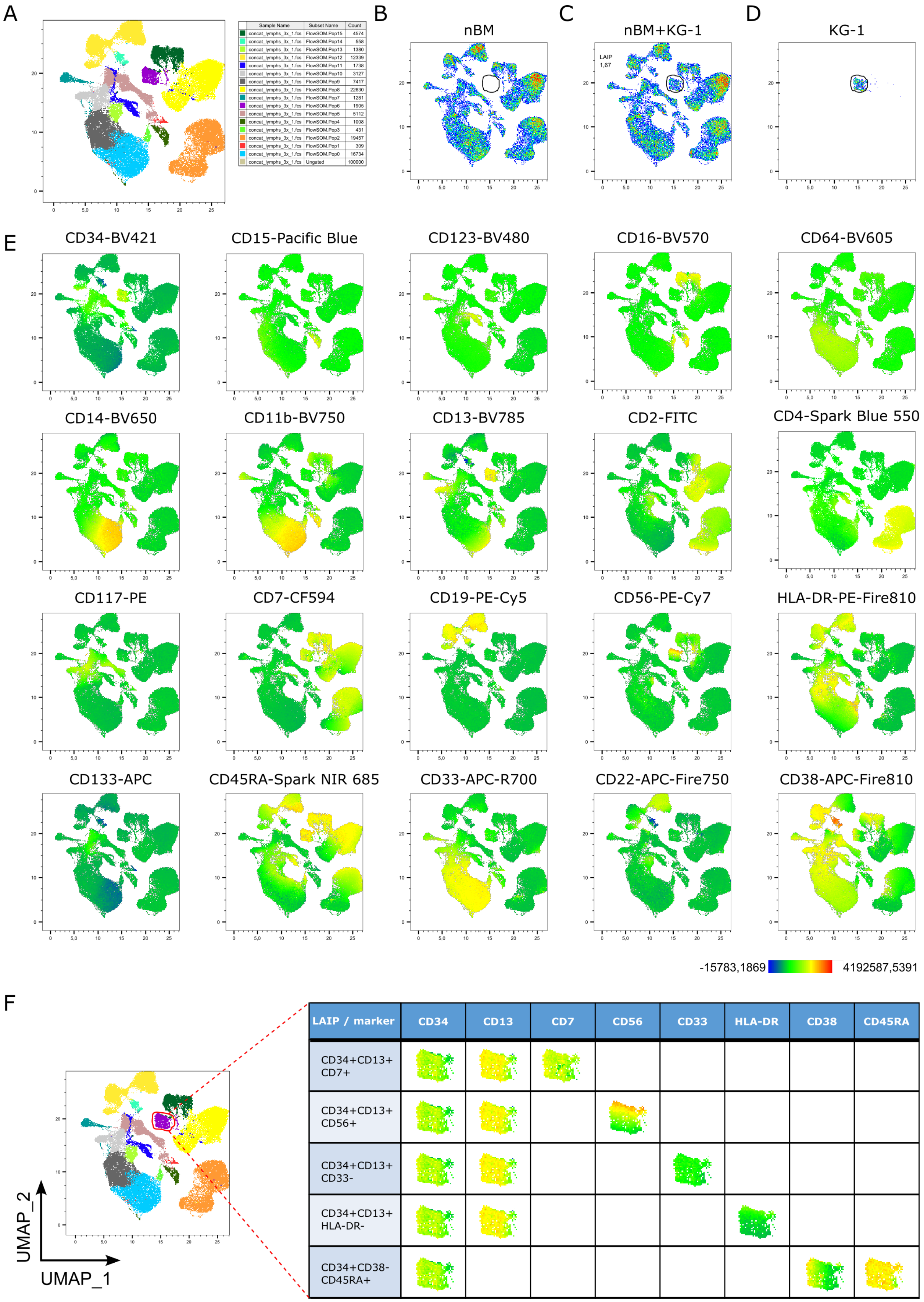

Figure S7: Identification of KG-1 cells and KG-1 derived LAIPs within nBM.
A mixture of nBM and KG-1 cells as well as pure nBM and KG-1 cells were stained with the 22-color panel and assessed by dimensional reduction and clustering for the identification of KG-1 cells. (**A**) UMAP representation of a mixed nBM + KG-1 cell sample with color-coded FlowSOM clusters. (**B**) UMAP of pure nBM. (**C**) UMAP of nBM mixed with KG-1 cells. (**D**) UMAP of pure KG-1 cells. (**E**) Heat-map representation of marker expression profiles for the UMAP of nBM mixed with KG-1 cells. (CD45⁺ and viability⁺ were gated prior to clustering and are therefore not included). (**F**) Heat-map representation of KG-1 derived LAIP markers.

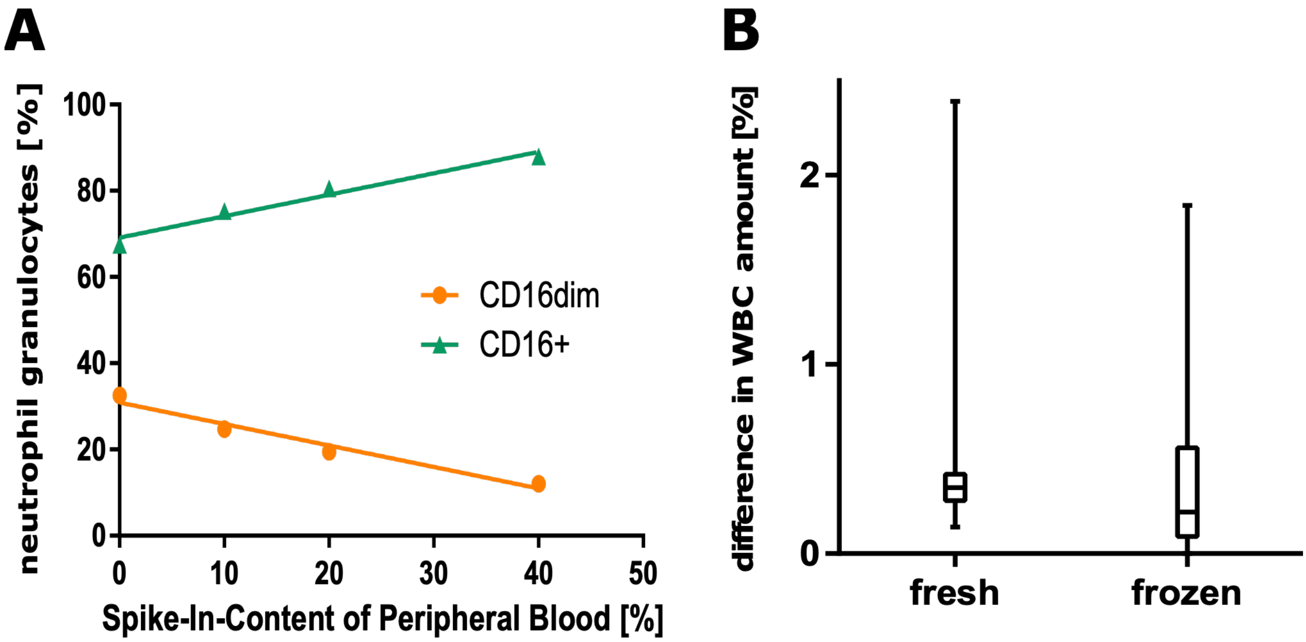

Figure S8: Hemodilution tracking through CD16 and viability marker assessment
(A) For the same samples as shown in Figure 3 the size of the $CD16^{+}$ and $CD16^{dim}$ populations is shown as a function of increasing proportions of PB. **(B)** Difference in WBC amount when gating a 22-color sample with and without the viability marker (Zombie NIR), for measured samples from fresh (n= 39) and frozen (n= 18) material.

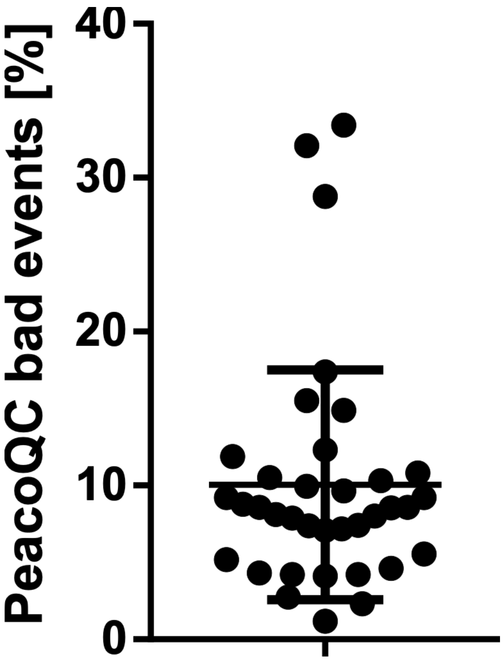

Figure S9: Removed events via PeacoQC.
The samples from the KG-1 dilution experiments for LAIP-assessment (n=36 fresh samples) were measured on the Cytek Northern Lights CLC with the 22-color panel and subsequently cleaned via PeacoQC, which was run as a FlowJo Plugin. The graph shows single data points, as well as mean and standard deviation ranges for the percentage of removed events via PeacoQC (10.1 ± 7.5%).

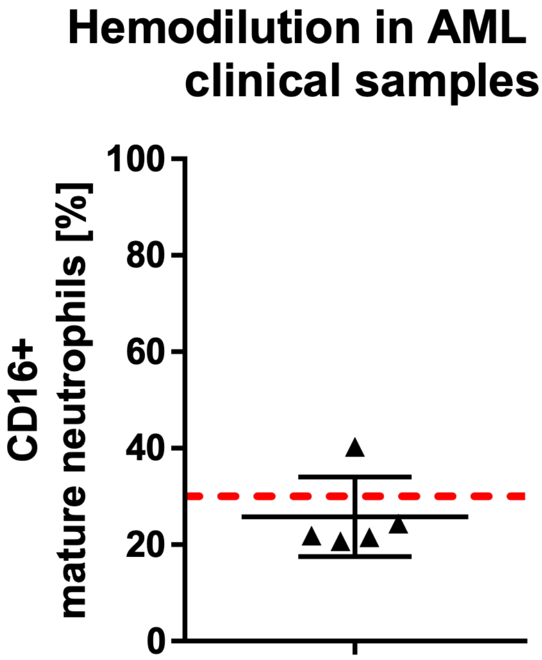

Figure S10: Hemodilution in AML MRD clinical samples

AML MRD samples were measured with the 22-color panel on the Cytek NL CLC machine and analyzed in Infinicyt through the gating strategy depicted in Figure 2. An analysis of the $CD16^+$ mature neutrophil population was performed. The box-plot shows the percentages of $CD16^+$ mature neutrophils in the corresponding AML MRD samples (mean and standard deviation included as straight lines). The red dotted line represents the proposed quantitative threshold for hemodilution from Loken et al. (https://pubmed.ncbi.nlm.nih.gov/18548614/), which is achieved at 30% $CD16^+$ mature neutrophils.
